# Rapid detection and strain typing of *Chlamydia trachomatis* using a highly multiplexed microfluidic PCR assay

**DOI:** 10.1371/journal.pone.0178653

**Published:** 2017-05-31

**Authors:** Rosemary S. Turingan, Ludmila Kaplun, Greice Krautz-Peterson, Sarah Norsworthy, Anna Zolotova, Sandeep J. Joseph, Timothy D. Read, Deborah Dean, Eugene Tan, Richard F. Selden

**Affiliations:** 1 NetBio, Waltham, Massachusetts, United States of America; 2 Department of Medicine, Division of Infectious Diseases and Department of Human Genetics, Emory University School of Medicine, Atlanta, Georgia, United States of America; 3 Center for Immunobiology and Vaccine Development, Children’s Hospital Oakland Research Institute, Oakland, California, United States of America; 4 University of California at San Francisco School of Medicine, San Francisco, California, United States of America; 5 University of California at Berkeley and University of California at San Francisco Joint Graduate Program in Bioengineering, Berkeley, California, United States of America; Universita degli Studi di Bologna, ITALY

## Abstract

Nucleic acid amplification tests (NAATs) are recommended by the CDC for detection of *Chlamydia trachomatis* (*Ct*) urogenital infections. Current commercial NAATs require technical expertise and sophisticated laboratory infrastructure, are time-consuming and expensive, and do not differentiate the lymphogranuloma venereum (LGV) strains that require a longer duration of treatment than non-LGV strains. The multiplexed microfluidic PCR-based assay presented in this work simultaneously interrogates 13 loci to detect *Ct* and identify LGV and non-LGV strain-types. Based on amplified fragment length polymorphisms, the assay differentiates LGV, ocular, urogenital, and proctocolitis clades, and also serovars L_1_, L_2,_ and L_3_ within the LGV group. The assay was evaluated in a blinded fashion using 95 clinical swabs, with 76 previously reported as urogenital *Ct*-positive samples and typed by *omp*A genotyping and/or Multi-Locus Sequence Typing. Results of the 13-plex assay showed that 51 samples fell within urogenital clade 2 or 4, 24 samples showed both clade 2 and 4 signatures, indicating possible mixed infection, gene rearrangement, or inter-clade recombination, and one sample was a noninvasive trachoma biovar (either a clade 3 or 4). The remaining 19 blinded samples were correctly identified as LGV clade 1 (3), ocular clade 3 (4), or as negatives (12). To date, no NAAT assay can provide a point-of-care applicable turnaround time for *Ct* detection while identifying clinically significant *Ct* strain types to inform appropriate treatment. Coupled with rapid DNA processing of clinical swabs (approximately 60 minutes from swab-in to result-out), the assay has significant potential as a rapid POC diagnostic for *Ct* infections.

## Introduction

*Chlamydia trachomatis (Ct)* is a Gram-negative obligate intracellular pathogen responsible for over 110 million global sexually transmitted disease (STD) cases annually, with sequelae including pelvic inflammatory disease (PID), ectopic pregnancy, and infertility [1, 2]. *Ct* is also responsible for outbreaks of lymphogranuloma venereum (LGV) and trachoma, a chronic ocular disease that can lead to blindness [3, 4].

Screening for *Ct* infections is an important means for reducing sequelae and interrupting transmission, especially because asymptomatic infections are so common among both males and females. A 2014 U.S. preventive services task force (USPSTF) report recommended screening for *Ct* in all sexually active females aged 24 years or younger and in older women who are at increased risk for infection [5]. Routine screening for *Ct* infection was not recommended in all sexually active young men but should be considered in adolescent and STD clinics, correctional facilities, and in populations with a high burden of infection (e.g., men who have sex with men; MSM) [6].

Current diagnostics for *Ct* are focused on nucleic acid amplification tests (NAATs), which are recommended by the CDC as they provide the highest sensitivity of available detection approaches [6, 7]. There are six NAAT assays that have been cleared by the FDA for *Ct* detection. Each of these tests uses only one or two *Ct* loci for detection, leading to the possibility of false negative results due to mutational or recombinational events that eliminate specific target binding sequences. For example, two NAATs missed detection of a Swedish *Ct* strain variant containing a deletion at a primer binding site [8]. This led to a significant increase in *Ct* STIs in Sweden and other parts of Europe [8]. Furthermore, even with the advent of NAATs, there is a long interval between specimen collection and generation of test findings. This turnaround time prevents diagnosis and appropriate treatment at the point-of-care (POC), and can result in patient loss to follow-up [9, 10]. Early diagnosis of infection is essential not only for the health of the individual patient but also for prevention of further transmission in the community. Unfortunately, the low sensitivity of currently available nonNAAT-based POC tests for *Ct* (25–65%) prevent their widespread use and indicate an urgent need for the development of a more sensitive POC assay [11].

*Ct* consists of 18 serological variants (serovars) with a number of subvariants that are identified based on serological reactivity of the epitopes of the major outer membrane protein (MOMP) [12, 13]. These serovars belong to two biological variants (biovars) with distinctive clinical infections: 1) lymphogranuloma venereum (LGV; clade 1), which includes serovars L_1_, L_2_, and L_3_ causes invasive STDs; and 2) the noninvasive trachoma epitheliotropic biovar, which is divided into ocular and urogenital subcategories (clades 2, 3, and 4) [12, 14]. The four *Ct* clades have been defined based on whole genome sequencing studies [15, 16]. Sequencing the *ompA* gene that encodes MOMP in addition to Multi-Locus Sequence Typing (MLST) have refined *Ct* typing, identifying significantly more strains than has been possible with serotyping [17–19]. Among noninvasive *Ct* strains, ocular serovars A, B, Ba, and C (clade 3) cause blinding trachoma while D, Da, E, F, G, H, I, Ia, J, Ja and K [20] cause urogenital pathologies including PID and infertility. The urogenital strains are divided into two clades (clade 2 and clade 4), the latter having been shown to be involved in the development of proctocolitis.

Since 2003, LGV outbreaks have been reported in Europe, Australia, New Zealand, the United States, and Canada [21] causing lymphatic obstruction and ulcerative proctitis; recent outbreaks predominantly found among HIV positive MSM have been associated with the L_2_b subserovar [21]. LGV infection requires a 21-day course of antibiotics, whereas the non-LGV urogenital and rectal *Ct* infections can be effectively treated with a one- to seven- day course of the same antibiotics while ocular trachoma can be treated with a single oral dose of azithromycin or with tetracycline eye ointment. Despite the specific clinical outcomes associated with particular *Ct* lineages, none of the commercially available *Ct* detection assays allow strain type differentiation for determining the appropriate course of treatment [6, 22–24].

We have previously developed a 9-plex microfluidic PCR for POC detection of *Ct* and demonstrated that it has higher sensitivity and specificity than a commercial NAAT [25]. Here, we present an enhanced 13-plex assay that now incorporates *Ct* genotyping capabilities, targeting 11 chromosomal and 2 cryptic plasmid located loci. The combination of multiple targets in both plasmid and chromosome loci decreases the likelihood of generating false negative results from mutation or recombination [8]. The 13-plex rapid microfluidic PCR assay described here enables rapid differentiation of clinically important biovars, clades, and strains based on an approximately 22-minute PCR amplification reaction yielding characteristic electrophoretic signatures for the various *Ct* strain types.

## Materials and methods

### DNA isolates

Purified DNA from 37 previously sequenced *Ct* reference strains and other urovaginal pathogens and commensals were obtained from ATCC (Manassas, VA): *Ct* strains A/Har-13 (VR-571B), B-Har-36 (VR-573), D/UW-3/Cx (VR-885D), E/Bour (VR-348BD), F/IC-Cal-3 (VR-346), G/UW-57/Cx (VR-878D), H/UW-43/Cx (VR-879D), J/UW-36/Cx (VR-886D), K/UW-31/Cx (VR-887), L1/440 (VR-901B), L2/T'ang (VR-577), L2/434 (VR-902DB), L3/404 (VR-903D), *Neisseria gonorrhoeae*, strain SK-92-679 (BAA-1846CD), *Neisseria lactamica* (23970), *Neisseria flava* (14221D), *Neisseria perflava* (14799D-5), G*ardnerella vaginalis*, strain AmMS 117 (49145D-5), *Trichomonas vaginalis*, *strain C-1* (30001D), *Enterococcus faecium*, strain MMC4 (51559D-5), *Enterococcus faecalis*, strain V583 (700802-D) and *Candida albicans* (MYA-2876). Twenty-five additional DNAs from *Ct* strains and clinical isolates [15, 26] were provided by Dr. Dean.

Eighty-eight DNAs were purified from endocervical swabs collected from 15 to 24 year old high risk female patients at STD clinics in the San Francisco Bay Area and identified as *Ct* positive or negative based on the Roche Amplicor NAAT (Roche Diagnostics, Indianapolis, IN) test and NetBio’s previously described 9-plex assay based on 7 MLST targets, *omp*A, and the cryptic plasmid [25]. Seven additional clinical samples blinded to NetBio were provided by Dr. Dean from her DNA collection. These samples had been previously typed using both *omp*A genotyping and MLST. Human DNA was purified from fresh whole blood. All clinical samples were remnant samples not specifically collected for this study. Because the samples were remnants and de-identified with no trace back to the patients, the UCSF Benioff Children's Hospital IRB does not consider this work human subjects research.

### DNA purification

The eighty-eight clinical swabs were purified using a guanidinium-based lysis solution and following a silica-based bind-wash-elute protocol as previously described [25]. DNA from seven additional endocervical swab samples were purified using the High Pure Kit (Roche) as previously described [27]. Purified DNAs were quantified using a Nanodrop spectrophotometer (Thermo Scientific NanoDrop Products, Wilimington, DE).

### PCR primer design

Eighty-one published sequencing assemblies with full *Ct* genomes and 67 with partial genomes available through the NCBI assembly database, as well as full genome sequencing data from an additional 32 commercial strains and clinical isolates (some of which were submitted to SRA database) were utilized to locate appropriate regions for primers selection. The list of *Ct* genomes used in the alignment is provided in S1 Table (Supporting information). The major criteria were conserved regions for *Ct* detection and resulting amplicons with variable length regions for differentiation. Commensal species and additional urogenital pathogens (i.e. *Chlamydiaceae*, yeast, *G*. *vaginalis*, *T*. *vaginalis*, and *Enterococcus*) that may be present in clinical specimens as well as host (*H*. *sapiens) genomic DNA* were also included in the *in silico* analysis to allow design of *Ct*-specific primers. Sequence search was performed by blastn algorithm directly through NCBI web interface and through Geneious7.1 software (http://blast.ncbi.nlm.nih.gov/Blast.cgi) [28] (http://www.geneious.com) [29] using reference sequences from LGV strain L2/434/Bu. Sequences were aligned by Geneious and by ClustalW algorithms through Geneious 7.1 software (http://www.geneious.com) [29]. Primer thermodynamic properties and their compatibility and specificity were verified using VisualOMP software version 7.8.42.0 (http://www.dnasoftware.com/). Primers for all loci and all genotypic variants were designed such that the amplicons were distinguishable by size. Data on the resolution of the separation and detection (S&D) system has been previously reported and showed single base resolution across 100–500 base sizing range [30, 31]. Fluorescent labels were placed at the 5’ end of one primer of each pair. Four fluorescent labels were utilized: FAM, ROX, TMR and JOE. Sequences of primers for each target are provided in S2 Table (Supporting information).

### Microfluidic multiplex PCR amplification and separation

The microfluidic biochip and rapid thermal cycler were used for amplification of multiplexed PCR [32, 33]. For *Ct* detection and genotyping, a 7 μl reaction mixture was amplified in 22 minutes essentially as described [33]. Amplicons were microfluidically separated and detected by laser-induced fluorescence using the Genebench-FX instrument and accompanying biochip, also as described [33].

### Microfluidic Sanger sequencing

Select PCR amplicons were subjected to microfluidic Sanger sequencing as previously described [34]. PCR products were directly used as template for microfluidic sequencing reactions using the BigDye^®^ Terminator v3.1 cycle sequencing kit (Applied Biosystems, Foster City, CA).

### Multiplex assay development and testing

Primer concentrations in the multiplex assay were adjusted to produce approximately equal signal strength of all peaks in each fluorescent channel to optimize analysis. By design, multi-copy targets such as those located in the plasmid and ribosomal regions were placed in the FAM channel to achieve an optimal limit of detection. In addition to primers designed to detect *Ct* targets, an MS2 phage internal control (primer pair and DNA target) allows monitoring of PCR reaction performance.

During assay development and validation, 100 genome equivalents of purified *Ct* DNAs (from select sequenced *Ct* reference strains) were used for amplification. For evaluation of assay sensitivity, 10 and 1 genomic equivalents of DNA were tested. Primer pair cross-reactivity against host DNA was tested using 10,000 human genome equivalents per reaction and against DNAs from commensal and pathogenic species using 100,000 genome equivalents of each species.

To evaluate performance of the assay using clinical specimens containing background DNAs (including from humans and microorganisms), a total of 95 clinical samples were tested in a blinded fashion. Purified DNA (10-40ng) from seventy-six endocervical clinical swab samples previously identified [25] as “true” positives, 12 representative samples identified as “true” negatives for *Ct* infection, and an additional seven clinical samples previously characterized as having LGV or ocular infection was tested. All assays were performed in triplicate.

### Nomenclature

The amplicon size corresponding to each *Ct* target was reported as **a** fragment length type (FLT). FLTs were assigned lower case Roman numerals i-v (Table 1). The combined FLTs from all loci were reported as high resolution amplified fragment length polymorphisms (hAFLP), and hAFLPs were assigned Arabic numerals (Tables 2 and 3).

**Table 1:** *Ct* amplification targets and their strain types based on expected and observed amplicon sizes.

		Invasive				Non-invasive															
	Disease Phenotype	LGV (clade 1)				Ocular trachoma (clade 3)				Urogenital/Proctocolitis (clade 4)							Urogenital (clade 2)				
	Serovar/Subserovar	L3	L1	L2	L2b	A	B	Ba	C	I	K	D	H	G	Ia	J	D	E	F	Ja	Da
Target	Target copy number	Amplicon size in bases (FLT)				Amplicon size in bases (FLT)				Amplicon size in bases (FLT)											
***p*CT8**	**0–8 copies**	**197 (FLT i)**				**197 (FLT i)**				**197 (FLT i)**											
***u16S***	**2 copies**	**238 (FLT i)**				**238 (FLT i)**				**238 (FLT i)**											
***23S_5S***	**2 copies**	**244 (FLT i)**				**244 (FLT i)**				**244 (FLT i)**											
***p*CT7**	**0–8 copies**	**260 (FLT i)**				**260 (FLT i)**				**260 (FLT i)**											
**IGS-101**	**1 copy**	**207 (FLT i)**				**320 (FLT ii)**				**320 (FLT ii)**											
**IGS-102**	**1 copy**	**329 (FLT i)**				**315 (FLT ii)**				**315 (FLT ii)**							**329 (FLT i)**				
**IGS-103**	**1 copy**	**350 (FLT i) 351 (FLT iv)**				**228 (FLT ii); 229 (FLT iii)**				**351 (FLT iv) 352 (FLT v)**											
***mdh*C**	**1 copy**	**413 (FLT i)**				**413 (FLT i)**				**413 (FLT i)**											
**IGS-104**	**1 copy**	**284 (FLT i)**			**293 (FLT iii)**	**289 (FLT ii)**				**284 (FLT i)**											**289 (FLT ii)**
**IGS-105**	**1 copy**	**349 (FLT i)**				**331 (FLT ii); 332 (FLT iii)**				**331 (FLT ii); 332 (FLT iii)**											
***omp*A**	**1 copy**	**n/a**	**379 (FLT ii)**	**381 (FLT i)**		**n/a**				**n/a**											
**IGS-106**	**1 copy**	**403 (FLT i)**				**403 (FLT i)**				**403 (FLT i)**							**400 (FLT ii)**				
**IGS-107**	**1 copy**	**418 (FLT i)**				**418 (FLT i)**				**264 (FLT ii); 420 (FLT iii)**											
**Internal control**		**280**																			

Targets are color-coded to reflect the fluorescent dye used for detection—FAM, blue; TAMRA, yellow; ROX, red; JOE, green. The expected and observed amplicons sizes are indicated in bases and assigned a fragment length type (FLT).

^***a***^n/a, Not Applicable as target copy or FLT not expected.

**Table 2:** Evaluation of the *Ct* 13-plex amplification assay for the detection and strain typing of 37 previously-sequenced *Ct* reference strains.

			Target/FLT													*Ct* 13-plex Results		
Sample No	*Ct* Strain	Previously Assigned Clade	pCT8	u16S	23S_5S	pCT7	IGS-101	IGS-102	IGS-103	*mdh*C	IGS-104	IGS-105	ompA	IGS-106	IGS-107	hAFLP type	No of Targets detected	Clinical Classification based on hAFLP type
**1**	**L2/434 [AM884176]**	**1**	**i**	**i**	**i**	**i**	**i**	**i**	**i**	**i**	**i**	**i**	**i**	**i**	**i**	**1**	**13**	**LGV/L2**
**2**	**L3/404 [ASM31910]**	**1**	**i**	**i**	**i**	**i**	**i**	**i**	**iv**	**i**	**i**	**i**	**n/a**^b^	**i**	**i**	**2**	**12**	**LGV/L3**
**3**	**L1/440 [ASM31882]**	**1**	**i**	**i**	**i**	**i**	**i**	**i**	**iv**	**i**	**i**	**i**	**ii**	**i**	**i**	**3**	**13**	**LGV/L1**
**4**	**L2/T'ang**	**1**	**i**	**i**	**i**	**i**	**i**	**i**	**iv**	**i**	**i**	**i**	**i**	**i**	**i**	**4**	**13**	**LGV/L2**
**5**	**Clinical L2a**	**1**	**i**	**i**	**i**	**i**	**i**	**i**	**iv**	**i**	**i**	**i**	**i**	**i**	**i**	**4**	**13**	**LGV/L2**
**6**	**Clinical L2c**	**1**	**i**	**i**	**i**	**i**	**i**	**i**	**iv**	**i**	**i**	**i**	**i**	**i**	**i**	**4**	**13**	**LGV/L2**
**7**	**L2b/48nl**	**1**	**i**	**i**	**i**	**i**	**i**	**i**	**iv**	**i**	**i**	**i**	**i**	**i**	**i**	**4**	**13**	**LGV/L2**^d^
**8**	**I/UW-12/UR**	**4**	**i**	**i**	**i**	**i**	**i**	**i**	**iv**	**i**	**i**	**i**	**i**	**i**	**i**	**4**	**13**	**LGV/L2**^e^
**9**	**H/UW-43/Cx [ERS011032]**	**4**	**i**	**i**	**i**	**i**	**ii**	**ii**	**v**	**i**	**i**	**iii**	**n/a**^b^	**i**	**ii**	**5**	**12**	**Urogenital/Proctocolitis**
**10**	**D/UW-3/Cx [AE001273]**	**4**	**i**	**i**	**i**	**i**	**ii**	**ii**	**v**	**i**	**i**	**iii**	**n/a**^b^	**i**	**ii**	**5**	**12**	**Urogenital/Proctocolitis**
**11**	**G/UW-57/Cx [SRA051445.1]**	**4**	**i**	**i**	**i**	**i**	**ii**	**ii**	**v**	**i**	**i**	**iii**	**n/a**^b^	**i**	**ii**	**5**	**12**	**Urogenital/Proctocolitis**
**12**	**K/UW-31/Cx [ERS011035]**	**4**	**i**	**i**	**i**	**i**	**ii**	**ii**	**v**	**i**	**i**	**iii**	**n/a**^b^	**i**	**ii**	**5**	**12**	**Urogenital/Proctocolitis**
**13**	**J/UW-36/Cx [ERS11034]**	**4**	**i**	**i**	**i**	**i**	**ii**	**ii**	**v**	**i**	**i**	**iii**	**n/a**^b^	**i**	**ii**	**5**	**12**	**Urogenital/Proctocolitis**
**14**	**Ja/UW-92**	**4**	**i**	**i**	**i**	**i**	**ii**	**ii**	**v**	**i**	**i**	**iii**	**n/a**^b^	**i**	**ii**	**5**	**12**	**Urogenital/Proctocolitis** ^f^
**15**	**Clinical Ia**	**4**	**i**	**i**	**i**	**i**	**ii**	**ii**	**v**	**i**	**i**	**iii**	**n/a**^b^	**i**	**ii**	**5**	**12**	**Urogenital/Proctocolitis**
**16**	**H/UW-4/Cxa [SRA051548.1]**	**4**	**i**	**i**	**i**	**i**	**ii**	**ii**	**v**	**i**	**i**	**iii**	**n/a**^b^	**i**	**ii**	**5**	**12**	**Urogenital/Proctocolitis**
**17**	**Clinical J**	**4**	**i**	**i**	**i**	**i**	**ii**	**ii**	**v**	**i**	**i**	**iii**	**n/a**^b^	**i**	**ii**	**5**	**12**	**Urogenital/Proctocolitis**
**18**	**K/187l**	**4**	**i**	**i**	**i**	**i**	**ii**	**ii**	**v**	**i**	**i**	**iii**	**n/a**^b^	**i**	**ii**	**5**	**12**	**Urogenital/Proctocolitis**
**19**	**E/Bour [ASM31864v1]**	**2**	**i**	**i**	**i**	**i**	**ii**	**i**	**v**	**i**	**i**	**iii**	**n/a**^b^	**ii**	**ii**	**6**	**12**	**Urogenital**
**20**	**F/IC-Cal-3 [ERS011030]**	**2**	**i**	**i**	**i**	**i**	**ii**	**i**	**v**	**i**	**i**	**iii**	**n/a**^b^	**ii**	**ii**	**6**	**12**	**Urogenital**
**21**	**Clinical D**	**NA**^c^	**i**	**i**	**i**	**i**	**ii**	**i**	**v**	**i**	**i**	**iii**	**n/a**^b^	**ii**	**ii**	**6**	**12**	**Urogenital**
**22**	**Clinical D**	**NA**^c^	**i**	**i**	**i**	**i**	**ii**	**i**	**v**	**i**	**i**	**iii**	**n/a**^b^	**ii**	**ii**	**6**	**12**	**Urogenital**
**23**	**D/199nl**	**NA**^c^	**i**	**i**	**i**	**i**	**ii**	**i**	**v**	**i**	**i**	**iii**	**n/a**^b^	**ii**	**ii**	**6**	**12**	**Urogenital**
**24**	**E/39nl**	**2**	**i**	**i**	**i**	**i**	**ii**	**i**	**v**	**i**	**i**	**iii**	**n/a**^b^	**ii**	**ii**	**6**	**12**	**Urogenital**
**25**	**D2/1891**	**NA**^c^	**i**	**i**	**i**	**i**	**ii**	**i**	**v**	**i**	**i**	**iii**	**n/a**^b^	**ii**	**ii**	**6**	**12**	**Urogenital**
**26**	**Ja/47nl [SRA051542.1]**	**2**	**i**	**i**	**i**	**i**	**ii**	**i**	**v**	**i**	**i**	**iii**	**n/a**^b^	**ii**	**ii**	**6**	**12**	**Urogenital**
**27**	**Clinical E**	**2**	**i**	**i**	**i**	**i**	**ND**^a^	**i**	**v**	**i**	**ND**^a^	**ND**^a^	**n/a**^b^	**ii**	**ii**	6*	**9**	**Urogenital**
**28**	**Clinical E**	**2**	**i**	**i**	**i**	**i**	**ND**^a^	**i**	**v**	**i**	**i**	**iii**	**n/a**^b^	**ii**	**ND**^a^	6*	**10**	**Urogenital**
**29**	**A/Har-13 [CP000051]**	**3**	**i**	**i**	**i**	**i**	**ii**	**ii**	**iii**	**i**	**ii**	**iii**	**n/a**^b^	**i**	**i**	**7**	**12**	**Ocular trachoma**
**30**	**Da/TW-448**	**NA**^c^	**i**	**i**	**i**	**i**	**ii**	**i**	**v**	**i**	**ii**	**iii**	**n/a**^b^	**ii**	**ii**	**8**	**12**	**Urogenital/Da**
**31**	**Clinical E**	**2**	**i**	**i**	**i**	**i**	**ii**	**i**	**v**	**i**	**i**	**iii**	**n/a**^b^	**ii**	**iii**	**9**	**12**	**Urogenital**
**32**	**D/210n**	**NA**^c^	**i**	**i**	**i**	**i**	**ii**	**i**	**v**	**i**	**i**	**iii**	**n/a**^b^	**ii**	**iii**	**9**	**12**	**Urogenital**
**33**	**Ba/Apache-2**	**3**	**i**	**i**	**i**	**i**	**ii**	**ii**	**ii**	**i**	**ii**	**iii**	**n/a**^b^	**i**	**i**	**10**	**12**	**Ocular trachoma**
**34**	**C/TW-3/OT [SRA051538.1]**	**3**	**i**	**i**	**i**	**i**	**ii**	**ii**	**ii**	**i**	**ii**	**iii**	**n/a**^b^	**i**	**i**	**10**	**12**	**Ocular trachoma**
**35**	**B/TW5/OT**	**3**	**i**	**i**	**i**	**i**	**ND**^a^	**ii**	**ii**	**i**	**ii**	**iii**	**n/a**^b^	**i**	**i**	10*	**11**	**Ocular trachoma**
**36**	**B/HAR-36**	**3**	**i**	**i**	**i**	**i**	**ND**^a^	**ii**	**ii**	**i**	**ii**	**iii**	**n/a**^b^	**i**	**i**	10*	**11**	**Ocular trachoma**
**37**	**B-Har-36 (ATCC VR-573)**	**3**	**i**	**i**	**i**	**i**	**ND**^a^	**ii**	**iii**	**i**	**ii**	**ii**	**n/a**^b^	**i**	**i**	11*	**11**	**Ocular trachoma**

The combined FLTs from all loci are reported as high resolution amplified fragment length polymorphisms (hAFLP). hAFLP type assignments marked with asterisks (*) indicate the absence of at least one amplicon. Corresponding Genbank accession numbers are in brackets. Samples 7 and 8 were subjected to targeted resequencing, and the identification made by the 13-plex assay was found to be correct in both cases.

^*a*^ ND, Not Detected.

^*b*^ n/a, Not Applicable as FLT is not expected.

^*c*^ NA, Not Assigned

^*d*^Atypical L_2_b strain. Multiplex assay results were consistent with an LGV L_2_ strain and were confirmed by Sanger sequencing.

^*e*^ Nonconcordant result due to tube labelling error. Multiplex assay results were consistent with an LGV L_2_ strain and were confirmed by Sanger sequencing.

^*f*^Atypical Ja strain. Multiplex assay results confirmed *in silico* sequence-based clade assignment.

**Table 3:** Detection and strain-typing of *Ct* DNA from 95 clinical samples using the *Ct* 13-plex assay.

	Target/FLT													*Ct* 13-plex Results		
Sample No	pCT8	u16S	23S_5S	pCT7	IGS-101	IGS-102	IGS-103	*mdh*C	IGS-104	IGS-105	ompA	IGS-106	IGS-107	hAFLP type	No of Targets detected	Clinical Classification based on hAFLP type
**1**	**i**	**i**	**i**	**i**	**ii**	**ii**	**v**	**i**	**i**	**iii**	**ND**^a^	**i**	**ii**	**5**	**12**	**Urogenital/Proctocolitis**
**2**	**i**	**i**	**i**	**i**	**ii**	**ii**	**v**	**i**	**i**	**iii**	**ND**^a^	**i**	**ii**	**5**	**12**	**Urogenital/Proctocolitis**
**3**	**i**	**i**	**i**	**i**	**ii**	**ii**	**v**	**i**	**i**	**iii**	**ND**^a^	**i**	**ii**	**5**	**12**	**Urogenital/Proctocolitis**
**4**	**i**	**i**	**i**	**i**	**ii**	**ii**	**v**	**i**	**i**	**iii**	**ND**^a^	**i**	**ii**	**5**	**12**	**Urogenital/Proctocolitis**
**5**	**i**	**i**	**i**	**i**	**ii**	**ii**	**v**	**i**	**i**	**iii**	**ND**^a^	**i**	**ii**	**5**	**12**	**Urogenital/Proctocolitis**
**6**	**i**	**i**	**i**	**i**	**ND**^a^	**ND**^a^	**ND**^a^	**ND**^a^	**ND**^a^	**iii**	**ND**^a^	**i**	**ND**^a^	**NI**^d^	**6**	**Urogenital/Proctocolitis or Ocular trachoma**
**7**	**i**	**i**	**i**	**i**	**ii**	**i**	**v**	**i**	**i**	**iii**	**ND**^a^	**ii**	**ii**	**6**	**12**	**Urogenital**
**8**	**i**	**i**	**i**	**i**	**ii**	**i**	**v**	**i**	**i**	**iii**	**ND**^a^	**ii**	**ii**	**6**	**12**	**Urogenital**
**9**	**i**	**i**	**i**	**i**	**ii**	**i**	**v**	**i**	**i**	**iii**	**ND**^a^	**ii**	**ii**	**6**	**12**	**Urogenital**
**10**	**i**	**i**	**i**	**i**	**ii**	**i**	**v**	**i**	**i**	**iii**	**ND**^a^	**ii**	**ii**	**6**	**12**	**Urogenital**
**11**	**i**	**i**	**i**	**i**	**ii**	**i**	**v**	**i**	**i**	**iii**	**ND**^a^	**ii**	**ii**	**6**	**12**	**Urogenital**
**12**	**i**	**i**	**i**	**i**	**ii**	**i**	**v**	**i**	**i**	**iii**	**ND**^a^	**ii**	**ii**	**6**	**12**	**Urogenital**
**13**	**i**	**i**	**i**	**i**	**ii**	**i**	**v**	**i**	**i**	**iii**	**ND**^a^	**ii**	**ii**	**6**	**12**	**Urogenital**
**14**	**i**	**i**	**i**	**i**	**ii**	**i**	**v**	**i**	**i**	**iii**	**ND**^a^	**ii**	**ii**	**6**	**12**	**Urogenital**
**15**	**i**	**i**	**i**	**i**	**ii**	**i**	**v**	**i**	**i**	**iii**	**ND**^a^	**ii**	**ii**	**6**	**12**	**Urogenital**
**16**	**i**	**i**	**i**	**i**	**ii**	**i**	**v**	**i**	**i**	**iii**	**ND**^a^	**ii**	**ii**	**6**	**12**	**Urogenital**
**17**	**i**	**i**	**i**	**i**	**ii**	**i**	**v**	**i**	**i**	**iii**	**ND**^a^	**ii**	**ii**	**6**	**12**	**Urogenital**
**18**	**i**	**i**	**i**	**i**	**ii**	**i**	**v**	**i**	**i**	**iii**	**ND**^a^	**ii**	**ii**	**6**	**12**	**Urogenital**
**19**	**i**	**i**	**i**	**i**	**ii**	**i**	**v**	**i**	**i**	**iii**	**ND**^a^	**ii**	**ii**	**6**	**12**	**Urogenital**
**20**	**i**	**i**	**i**	**i**	**ii**	**i**	**v**	**i**	**i**	**iii**	**ND**^a^	**ii**	**ii**	**6**	**12**	**Urogenital**
**21**	**i**	**i**	**i**	**i**	**ii**	**i**	**v**	**i**	**i**	**iii**	**ND**^a^	**ii**	**ii**	**6**	**12**	**Urogenital**
**22**	**i**	**i**	**i**	**i**	**ii**	**i**	**v**	**i**	**i**	**iii**	**ND**^a^	**ii**	**ii**	**6**	**12**	**Urogenital**
**23**	**i**	**i**	**i**	**i**	**ii**	**i**	**v**	**i**	**i**	**iii**	**ND**^a^	**ii**	**ii**	**6**	**12**	**Urogenital**
**24**	**i**	**i**	**i**	**i**	**ii**	**i**	**v**	**i**	**i**	**iii**	**ND**^a^	**ii**	**ii**	**6**	**12**	**Urogenital**
**25**	**i**	**i**	**i**	**i**	**ii**	**i**	**v**	**i**	**i**	**iii**	**ND**^a^	**ii**	**ii**	**6**	**12**	**Urogenital**
**26**	**i**	**i**	**i**	**i**	**ii**	**i**	**v**	**i**	**i**	**iii**	**ND**^a^	**ii**	**ii**	**6**	**12**	**Urogenital**
**27**	**i**	**i**	**i**	**i**	**ii**	**i**	**v**	**i**	**i**	**iii**	**ND**^a^	**ii**	**ii**	**6**	**12**	**Urogenital**
**28**	**i**	**i**	**i**	**i**	**ii**	**i**	**v**	**i**	**i**	**iii**	**ND**^a^	**ii**	**ii**	**6**	**12**	**Urogenital**
**29**	**i**	**i**	**i**	**i**	**ii**	**i**	**v**	**i**	**i**	**iii**	**ND**^a^	**ii**	**ii**	**6**	**12**	**Urogenital**
**30**	**i**	**i**	**i**	**i**	**ii**	**i**	**v**	**i**	**i**	**iii**	**ND**^a^	**ii**	**ii**	**6**	**12**	**Urogenital**
**31**	**i**	**i**	**i**	**i**	**ii**	**i**	**v**	**i**	**i**	**iii**	**ND**^a^	**ii**	**ii**	**6**	**12**	**Urogenital**
**32**	**i**	**i**	**i**	**i**	**ii**	**i**	**v**	**i**	**i**	**iii**	**ND**^a^	**ii**	**ii**	**6**	**12**	**Urogenital**
**33**	**i**	**i**	**i**	**i**	**ND**^a^	**i**	**v**	**ND**^a^	**ND**^a^	**iii**	**ND**^a^	**ND**^a^	**ii**	6*	**8**	**Urogenital**
**34**	**i**	**i**	**i**	**i**	**ND**^a^	**i**	**v**	**i**	**i**	**iii**	**ND**^a^	**ii**	**ii**	6*	**11**	**Urogenital**
**35**	**i**	**i**	**i**	**i**	**ND**^a^	**i**	**v**	**i**	**ND**^a^	**iii**	**ND**^a^	**ii**	**ii**	6*	**10**	**Urogenital**
**36**	**i**	**i**	**i**	**i**	**ii**	**i**	**v**	**ND**^a^	**i**	**iii**	**ND**^a^	**ii**	**ii**	6*	**11**	**Urogenital**
**37**	**i**	**i**	**i**	**i**	**ii**	**i**	**v**	**i**	**i**	**ND**^a^	**ND**^a^	**ii**	**iii**	9*	**11**	**Urogenital**
**38**	**i**	**i**	**i**	**i**	**ii**	**i**	**v**	**i**	**i**	**ND**^a^	**ND**^a^	**ii**	**iii**	9*	**11**	**Urogenital**
**39**	**i**	**i**	**i**	**i**	**ii**	**i**	**v**	**i**	**i**	**ND**^a^	**ND**^a^	**ii**	**iii**	9*	**11**	**Urogenital**
**40**	**i**	**i**	**i**	**i**	**ii**	**i**	**v**	**i**	**i**	**ND**^a^	**ND**^a^	**ii**	**iii**	9*	**11**	**Urogenital**
**41**	**i**	**i**	**i**	**i**	**ii**	**i**	**v**	**i**	**i**	**ND**^a^	**ND**^a^	**ND**^a^	**iii**	9*	**10**	**Urogenital**
**42**	**i**	**i**	**i**	**i**	**ii**	**i**	**v**	**i**	**i**	**ii**	**ND**^a^	**ii**	**ii**	**12**	**12**	**Urogenital**
**43**	**i**	**i**	**i**	**i**	**ii**	**i**	**v**	**i**	**i**	**ii**	**ND**^a^	**ii**	**ii**	**12**	**12**	**Urogenital**
**44**	**i**	**i**	**i**	**i**	**ii**	**i**	**v**	**i**	**i**	**ii**	**ND**^a^	**ii**	**ii**	**12**	**12**	**Urogenital**
**45**	**i**	**i**	**i**	**i**	**ii**	**i**	**v**	**i**	**i**	**ii**	**ND**^a^	**ii**	**ii**	**12**	**12**	**Urogenital**
**46**	**i**	**i**	**i**	**i**	**ii**	**ii**	**v**	**i**	**i**	**ii**	**ND**^a^	**i**	**ii**	**14**	**12**	**Urogenital/Proctocolitis**
**47**	**i**	**i**	**i**	**i**	**ii**	**ii**	**v**	**i**	**i**	**ii**	**ND**^a^	**i**	**ii**	**14**	**12**	**Urogenital/Proctocolitis**
**48**	**i**	**i**	**i**	**i**	**ii**	**i**	**iv**	**i**	**i**	**ND**^a^	**ND**^a^	**ii**	**iii**	18*	**11**	**Urogenital**
**49**	**ND**^a^	**i**	**ND**^a^	**i**	**ND**^a^	**i**	**v**	**i**	**i**	**ND**^a^	**ND**^a^	**ND**^a^	**ND**^a^	**NI**^d^	**6**	**Urogenital**
**50**	**i**	**i**	**i**	**i**	**ii**	**i**	**v**	**i**	**i**	**iii**	**ND**^a^	**ND**^a^	**ND**^a^	**NI**^d^	**10**	**Urogenital**
**51**	**i**	**i**	**i**	**i**	**ND**^a^	**i, iii**^e^	**v**	**ND**^a^	**i**	**ND**^a^	**ND**^a^	**i**	**ND**^a^	**NI**^d^	**8**	**Urogenital**
**52**	**i**	**i**	**i**	**i**	**ii**	**iii**^e^	**v**	**i**	**i**	**ND**^a^	**ND**^a^	**ND**^a^	**ND**^a^	**NI**^d^	**9**	**Urogenital**
**53**	**i**	**i**	**i**	**i**	**ii**	**iii**^e^	**iv**	**i**	**i**	**ND**^a^	**ND**^a^	**i**	**iii**	17*	**10**	**Urogenital and Urogenital/Proctocolitis**
**54**	**i**	**i**	**i**	**i**	**ii**	**iii**^e^	**v**	**i**	**i**	**ND**^a^	**ND**^a^	**i**	**iii**	13* or 16*	**10**	**Urogenital and Urogenital/Proctocolitis**
**55**	**i**	**i**	**i**	**i**	**ND**^a^	**iii**^e^	**v**	**i**	**i**	**ND**^a^	**ND**^a^	**i**	**iii**	13* or 16*	**9**	**Urogenital and Urogenital/Proctocolitis**
**56**	**i**	**i**	**i**	**i**	**ii**	**iii**^e^	**v**	**i**	**i**	**ND**^a^	**ND**^a^	**i**	**iii**	13* or 16*	**10**	**Urogenital and Urogenital/Proctocolitis**
**57**	**i**	**i**	**i**	**i**	**ii**	**i**	**iv**	**i**	**i**	**ii**	**ND**^a^	**i**	**iii**	**17**	**12**	**Urogenital and Urogenital/Proctocolitis**
**58**	**i**	**i**	**i**	**i**	**ii**	**i**	**v**	**i**	**i**	**iii**	**ND**^a^	**i**	**iii**	**13**	**12**	**Urogenital and Urogenital/Proctocolitis**
**59**	**i**	**i**	**i**	**i**	**ii**	**i**	**v**	**i**	**i**	**iii**	**ND**^a^	**i**	**iii**	**13**	**12**	**Urogenital and Urogenital/Proctocolitis**
**60**	**i**	**i**	**i**	**i**	**ii**	**i**	**v**	**i**	**i**	**iii**	**ND**^a^	**i**	**iii**	**13**	**12**	**Urogenital and Urogenital/Proctocolitis**
**61**	**i**	**i**	**i**	**i**	**ii**	**i**	**v**	**i**	**i**	**iii**	**ND**^a^	**i**	**iii**	**13**	**12**	**Urogenital and Urogenital/Proctocolitis**
**62**	**i**	**i**	**i**	**i**	**ii**	**i**	**v**	**i**	**i**	**iii**	**ND**^a^	**i**	**iii**	**13**	**12**	**Urogenital and Urogenital/Proctocolitis**
**63**	**i**	**i**	**i**	**i**	**ii**	**i**	**v**	**i**	**i**	**iii**	**ND**^a^	**i**	**ii**	**15**	**12**	**Urogenital and Urogenital/Proctocolitis**
**64**	**i**	**i**	**i**	**i**	**ii**	**i**	**v**	**i**	**i**	**iii**	**ND**^a^	**i**	**ii**	**15**	**12**	**Urogenital and Urogenital/Proctocolitis**
**65**	**i**	**i**	**i**	**i**	**ii**	**i**	**v**	**i**	**i**	**iii**	**ND**^a^	**i**	**ii**	**15**	**12**	**Urogenital and Urogenital/Proctocolitis**
**66**	**i**	**i**	**i**	**i**	**ii**	**i**	**v**	**i**	**i**	**iii**	**ND**^a^	**i**	**ii**	**15**	**12**	**Urogenital and Urogenital/Proctocolitis**
**67**	**i**	**i**	**i**	**i**	**ii**	**i**	**v**	**i**	**i**	**ii**	**ND**^a^	**i**	**iii**	**16**	**12**	**Urogenital and Urogenital/Proctocolitis**
**68**	**i**	**i**	**i**	**i**	**ii**	**i,iii**^e^	**v**	**i**	**i**	**iii**	**ND**^a^	**i, ii**	**ii, iii**	**19**	**12**	**Urogenital and Urogenital/Proctocolitis**
**69**	**i**	**i**	**i**	**i**	**ii**	**i,iii**^e^	**v**	**i**	**i**	**ND**^a^	**ND**^a^	**i, ii**	**iii**	20*	**11**	**Urogenital and Urogenital/Proctocolitis**
**70**	**i**	**i**	**i**	**i**	**ii**	**i**	**v**	**i**	**i**	**ii**	**ND**^a^	**i**	**ii, iii**	**21**	**12**	**Urogenital and Urogenital/Proctocolitis**
**71**	**i**	**i**	**i**	**i**	**ii**	**i**	**v**	**i**	**i**	**iii**	**ND**^a^	**i, ii**	**ii**	**22**	**12**	**Urogenital and Urogenital/Proctocolitis**
**72**	**i**	**i**	**i**	**i**	**ii**	**i**	**v**	**i**	**i**	**iii**	**ND**^a^	**i, ii**	**ii**	**22**	**12**	**Urogenital and Urogenital/Proctocolitis**
**73**	**i**	**i**	**i**	**i**	**ii**	**i**	**v**	**i**	**i**	**iii**	**ND**^a^	**i, ii**	**ii**	**22**	**12**	**Urogenital and Urogenital/Proctocolitis**
**74**	**i**	**i**	**i**	**i**	**ND**^a^	**i**	**v**	**i**	**i**	**iii**	**ND**^a^	**i, ii**	**ND**^a^	22*	**10**	**Urogenital and Urogenital/Proctocolitis**
**75**	**i**	**i**	**i**	**i**	**ii**	**i**	**iv**	**i**	**i**	**iii**	**ND**^a^	**i, ii**	**ii**	**23**	**12**	**Urogenital and Urogenital/Proctocolitis**
**76**	**i**	**i**	**i**	**i**	**ND**^a^	**i, ii**	**v**	**i**	**i**	**iii**	**ND**^a^	**ND**^a^	**ii**	24*	**10**	**Urogenital and Urogenital/Proctocolitis**
**77**	**ND**^a^	**ND**^a^	**ND**^a^	**ND**^a^	**ND**^a^	**ND**^a^	**ND**^a^	**ND**^a^	**ND**^a^	**ND**^a^	**ND**^a^	**ND**^a^	**ND**^a^	**n/a**^c^	**0**	***Ct*-negative**
**78**	**ND**^a^	**ND**^a^	**ND**^a^	**ND**^a^	**ND**^a^	**ND**^a^	**ND**^a^	**ND**^a^	**ND**^a^	**ND**^a^	**ND**^a^	**ND**^a^	**ND**^a^	**n/a**^c^	**0**	***Ct*-negative**
**79**	**ND**^a^	**ND**^a^	**ND**^a^	**ND**^a^	**ND**^a^	**ND**^a^	**ND**^a^	**ND**^a^	**ND**^a^	**ND**^a^	**ND**^a^	**ND**^a^	**ND**^a^	**n/a**^c^	**0**	***Ct-*negative**
**80**	**ND**^a^	**ND**^a^	**ND**^a^	**ND**^a^	**ND**^a^	**ND**^a^	**ND**^a^	**ND**^a^	**ND**^a^	**ND**^a^	**ND**^a^	**ND**^a^	**ND**^a^	**n/a**^c^	**0**	***Ct*-negative**
**81**	**ND**^a^	**ND**^a^	**ND**^a^	**ND**^a^	**ND**^a^	**ND**^a^	**ND**^a^	**ND**^a^	**ND**^a^	**ND**^a^	**ND**^a^	**ND**^a^	**ND**^a^	**n/a**^c^	**0**	***Ct-*negative**
**82**	**ND**^a^	**ND**^a^	**ND**^a^	**ND**^a^	**ND**^a^	**ND**^a^	**ND**^a^	**ND**^a^	**ND**^a^	**ND**^a^	**ND**^a^	**ND**^a^	**ND**^a^	**n/a**^c^	**0**	***Ct*-negative**
**83**	**ND**^a^	**ND**^a^	**ND**^a^	**ND**^a^	**ND**^a^	**ND**^a^	**ND**^a^	**ND**^a^	**ND**^a^	**ND**^a^	**ND**^a^	**ND**^a^	**ND**^a^	**n/a**^c^	**0**	***Ct-*negative**
**84**	**ND**^a^	**ND**^a^	**ND**^a^	**ND**^a^	**ND**^a^	**ND**^a^	**ND**^a^	**ND**^a^	**ND**^a^	**ND**^a^	**ND**^a^	**ND**^a^	**ND**^a^	**n/a**^c^	**0**	***Ct*-negative**
**85**	**ND**^a^	**ND**^a^	**ND**^a^	**ND**^a^	**ND**^a^	**ND**^a^	**ND**^a^	**ND**^a^	**ND**^a^	**ND**^a^	**ND**^a^	**ND**^a^	**ND**^a^	**n/a**^c^	**0**	***Ct-*negative**
**86**	**ND**^a^	**ND**^a^	**ND**^a^	**ND**^a^	**ND**^a^	**ND**^a^	**ND**^a^	**ND**^a^	**ND**^a^	**ND**^a^	**ND**^a^	**ND**^a^	**ND**^a^	**n/a**^c^	**0**	***Ct*-negative**
**87**	**ND**^a^	**ND**^a^	**ND**^a^	**ND**^a^	**ND**^a^	**ND**^a^	**ND**^a^	**ND**^a^	**ND**^a^	**ND**^a^	**ND**^a^	**ND**^a^	**ND**^a^	**n/a**^c^	**0**	***Ct-*negative**
**88**	**ND**^a^	**ND**^a^	**ND**^a^	**ND**^a^	**ND**^a^	**ND**^a^	**ND**^a^	**ND**^a^	**ND**^a^	**ND**^a^	**ND**^a^	**ND**^a^	**ND**^a^	**n/a**^c^	**0**	***Ct*-negative**
**89**	**i**	**i**	**i**	**i**	**ii**	**ii**	**iii**	**i**	**ii**	**iii**	**ND**^a^	**i**	**i**	**7**	**12**	**Ocular trachoma**
**90**	**i**	**i**	**i**	**i**	**i**	**i**	**i**	**i**	**i**	**i**	**i**	**i**	**i, ii**^b^	**1**	**13**	**LGV/L2**
**91**	**i**	**i**	**i**	**i**	**i**	**i**	**i**	**i**	**i**	**i**	**i**	**i**	**i**	**1**	**13**	**LGV/L2**
**92**	**i**	**i**	**i**	**i**	**ND**^a^	**ii**	**ii**	**i**	**ii**	**ii**	**ND**^a^	**i**	**i**	25*	**11**	**Ocular trachoma**
**93**	**i**	**i**	**i**	**i**	**i**	**i**	**iv**	**i**	**i**	**i**	**i**	**i**	**ii**^b^	**26**	**13**	**LGV/L2**
**94**	**i**	**i**	**i**	**i**	**ii**	**ii**	**iii**	**i**	**ii**	**iii**	**ND**^a^	**i**	**i**	**7**	**12**	**Ocular trachoma**
**95**	**i**	**i**	**i**	**i**	**ii**	**ii**	**iii**	**i**	**ii**	**iii**	**ND**^a^	**i**	**i**	**7**	**12**	**Ocular trachoma**

Identification was performed based on amplicon sizes as indicated by fragment length types (FLTs). The FLTs from all loci define the hAFLP type. hAFLP type assignments marked with asterisks (*) indicate the absence of at least one amplicon.

^*a*^ ND, Not Detected.

^*b*^ Peak suggesting a urogenital infection likely due to horizontal gene transfer at this locus.

^*c*^ n/a, Not Applicable as no FLTs are identified due to negative *Ct-*infection

^*d*^ NI, Not Identified due to several FLTs Not Detected

^*e*^FLT iii for IGS-102 is a new FLT variant due to tandem duplication of FLT i

## Results

### Rationale for target selection

Primers were designed for thirteen *Ct* targets to allow both pathogen detection and genotyping for intra-species differentiation of biovars and clades, and of clinically significant serovars (Table 1). Two *Ct* targets (*23S_5s* and *u16s*) are located within the ribosomal operon, present in two copies per genome, and two targets (*p*CT7 and *p*CT8) are located on the plasmid, present in up to eight copies within the majority of *Ct* strains [35].

Target *mdh*C represents a highly conserved housekeeping gene and was incorporated into the assay to ensure consistent recognition of *Ct*. Targets IGS-101 to IGS-107 are located in intergenic regions and were selected to allow strain-specific recognition. Primer pairs for these seven targets were designed to allow differentiation by amplicon size among *Ct* biogroups. Two of these targets (IGS-101 and IGS-105) are used for differentiation of the LGV biovar, while the remainder is used both for differentiation between ocular and urogenital trachoma and for differentiation between clades 2 and 4 within the urogenital trachoma biovar. Target *omp*A, within the highly variable major outer membrane gene, is used for L_1_-L_2_-L_3_ serogroup differentiation within the LGV biovar [36, 37]. The combination of results from IGS-104 and IGS-101/IGS-105 should allow differentiation of the L_2_b subserovar, the main etiologic agent of LGV cases currently spreading through developed countries [21]. Among urogenital strains, IGS-104 can also specifically discriminate strain Da. In addition to the thirteen *Ct* targets listed, the assay also utilizes an internal control amplifying spiked artificial template to allow evaluation of the overall assay performance.

The multiplex assay was designed to have back-ups for all targets, including both those for *Ct* detection and for biovar and clade differentiation. For detection purposes, u16s, 23s_5s, and *mdh*C are 3 ubiquitous targets allowing reliable *Ct* detection, while pCT7 and pCT8 are multi-copy loci that allow for potential improvement in the limit of detection (LOD) of the assay. In addition, 6 out of the 7 remaining targets yield *Ct*-specific amplicons. Clade differentiation is also accomplished using 2 targets, ensuring reliable clade detection even in the case of a target failure or mutation. Differentiation of the LGV biovar (clade1) from the trachoma biovar (clades 2, 3, and 4) relies on IGS-101 and IGS-105. Ocular clade 3 is differentiated from urogenital clades 2 and 4 by IGS-103 and IGS-107. Differentiation between clades 2 and 4 relies on IGS-102 and IGS-106. Finer differentiation leading to identification of serovars within clades relies on one target in each case (*omp*A for differentiation of serovars L_1_, L_2_ and L_3_ within LGV clade 1; IGS-104 for L_2_b subserovar identification within the L_2_ serovar, and for Da serovar differentiation within clade 2). Therefore, the only level of detection that might be compromised by mutation is the level of serovar differentiation; *Ct* detection and clade differentiation are ensured by the presence of multiple back-up targets. Targets and primers in this multiplex assay were selected and designed to provide differentiation of clades and only of certain serovars that generally have clinical implications. For example, the multiplex assay will not differentiate serovars I, K, D, H, G, Ia, and J within urogenital/proctocolitis clade 4; and L_2_a and L_2_c subserovars under clade 1 L_2_ serovar.

### Evaluation of *Ct* 13-plex microfluidic PCR assay sensitivity to simultaneously diagnose and strain type *Ct* strains

Thirty-seven previously sequenced *Ct* reference strains (samples 1 to 37 in Table 2) that had been characterized with respect to biovar/clade/strain were used to evaluate assay performance. The assay correctly identified all 37 *Ct* strains at 100 genomic equivalents per amplification reaction (Table 2). In addition to the correct clade detection for each one of these 37 strains, the assay was able to differentiate L_1_, L_2_, and L_3_ serovars within the LGV biovar, and Da serovar within the urogenital clade 2. The only available L_2_b isolate (sample 7) represents an atypical sequence (S3 Fig, Supporting information) that is different from the rest of L_2_b strains, which was then confirmed by Sanger sequencing. Thirty-two strains (86%) showed all twelve or thirteen FLTs, a result defined here as a “complete profile”; note that the o*mp*A locus does not amplify in some strains. Five strains (14%) generated partial profiles, missing 1–3 amplicons. As expected, all strains were positive for the multi-copy targets *p*CT8, *u16S*, *23S-5S*, and *p*CT7.

Six LGV strains identified by the 13-plex assay were concordant with labeled designations as provided. A seventh strain, previously identified as L_2_b/48nl was shown to be LGV L_2_ based on the presence of a 284bp fragment for the IGS-104 amplicon and a 381bp fragment for the *omp*A amplicon. Sequence analysis from Sanger reaction of the IGS-104 amplicon confirmed that the LGV L_2_ designation is correct. In addition to confirming amplicon sizing (a 9bp difference between LGV L_2_ and LGV L_2_b), sequencing revealed an A variant of the A/G adjacent SNP indicative of all LGV strains except L_2_b.

Twenty-nine strains were found to be from the noninvasive trachoma biovar. The sample previously identified as Strain I/UW-12/UR (sample 8) gave a nonconcordant result; the 13-plex assay identified it as LGV L_2_ with hAFLP 4 (Table 2) and not the expected hAFLP 5 for strains belonging to Serovar I. Sanger sequencing of the IGS-102 amplicon confirmed that the LGV L_2_ assignment of the sample is correct and that the tube was mislabeled. In addition to the size difference in IGS-102 amplicon (329bp, FLT i for LGV clade 1 versus 315bp, FLT ii for Serovar I clade 4), the LGV L_2_ designation shows a C variant of the C/T adjacent SNP typical for all LGVs but not in other *Ct* serovars (including Serovar I).

The assay also successfully identified all six ocular trachoma strains (clade 3) based on FLTs of targets IGS-103 (FLT ii or FLT iii corresponding to 228 and 229 bp respectively) and IGS-107 (FLT i corresponding to 418 bp). The remaining 23 strains were confirmed to be urogenital and further assessed for clade differentiation, i.e. clade 2 which is a group of strains causing urogenital disease only and clade 4, a group of strains that also causes proctocolitis. Thirteen strains were confirmed to be clade 2 and ten strains to be clade 4, based on the FLTs. Six clinical strains (D and Da/TW-448), for which no previous information about clade type was available, were identified as clade 2. Da/TW-448 was further identified as strain Da, due to the observed 289bp amplicon (FLT ii) at IGS-104 locus.

*In silico* sequence analyses showed a discrepancy between previously assigned strain identity and target gene sequences of two strains that may reflect recombination. In particular, sequences of strains Ja/UW-92 (sample 14) and J27-97 do not match the other Ja and J strains, respectively with respect to clades. This was confirmed by the results of the 13-plex for strain Ja/UW-92. Note that strain J27-97 was not available for multiplex testing but used for *in silico* analysis. Strain Ja/UW-92 was expected to have a 400bp fragment at IGS-106 for clade 2, but instead produced a 403bp fragment characteristic of strain J, belonging to clade 4. All the other Ja strains belong to clade 2. On the other hand, *in silico* alignment data focusing at IGS-102 and IGS-106 regions reveals that strain J27-97 belongs to clade 2; the other J strains belong to clade 4. Table 2 reflects correct classification of Ja/UW-92 based on the results of the multiplexed assay and as confirmed by sequence analysis.

**Fig 1:**
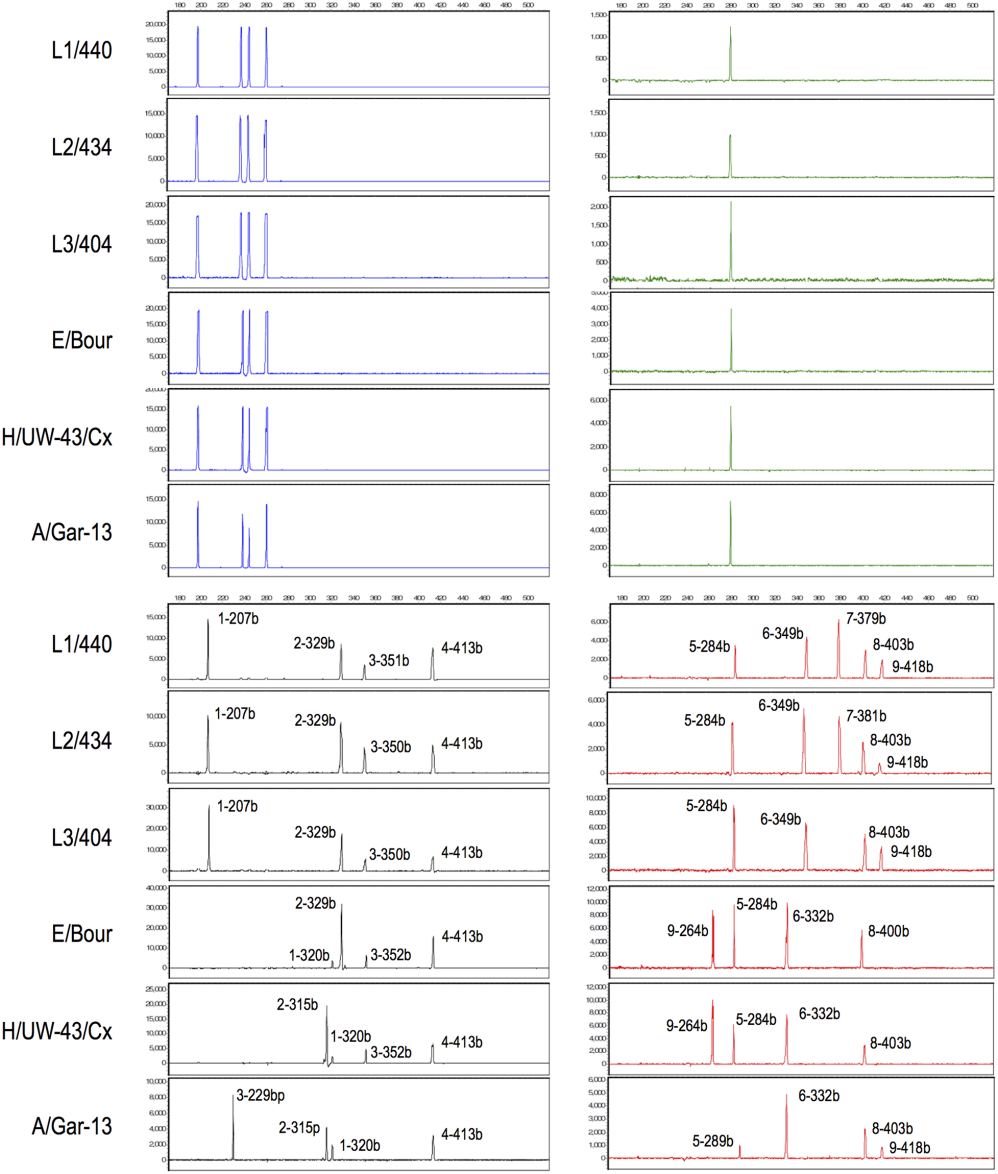
Generated 13-plex PCR electropherograms from six representative *Ct* strains: LGV strains L1/440, L2/434 and L3/404, urogenital strain E/Bour, proctocolitis strain H/UW-43/Cx and ocular strain A/Har-13. The FAM (blue), TMR (yellow but presented as black in the output profiles) and ROX (red) labeled-FLTs are aligned to illustrate amplicon sizes from the set of six strains. Targets and amplicon sizes in bases (b) are indicated to show variations observed among strains in the TMR and ROX channels (Peak 1 = IGS-101; Peak 2 = IGS-102; Peak 3 = IGS-103; Peak 4 = *mdh*C; Peak 5 = IGS-104; Peak 6 = IGS-105; Peak 7 = *omp*A; Peak 8 = IGS-106; and Peak 9 = IGS-107). Amplicon sizes in FAM are conserved and not numbered. The internal control amplicon is JOE-labeled (green). X-axis: fragment size in bases; Y-axis: relative fluorescence units (RFU).

Six representative, previously-sequenced reference strains having complete *Ct* profiles (13 peaks for strains L1/440 and L2/434 and 12 peaks for reference strains L3/404, E/Bour, H/UW-43/Cx and A/Har-13) are shown in Fig 1. The FAM-labeled multi-copy targets (blue peaks) were detected in all six strains. Targets IGS-101 in TMR (black peaks) and IGS-105 in ROX (red peaks) differentiate reference LGV strains (L1/440, L2/434 and L3/404) from the one trachoma and two urogenital strains based on characteristic FLTs. ROX-labelled *omp*A differentiates LGV strains (strain L1/440 (strain L_1_) has a FLT of 379 bp, L2/434 (strain L_2_) 381 bp, and strain L3/404 (strain L_3_) does not amplify *omp*A). IGS-103 (TMR) and IGS-107 (ROX) differentiate strain A/Har-13 (clade 3, ocular trachoma) from strains E/Bour (clade 2, urogenital) and H/UW-43/Cx (clade 4, proctocolitis). Finally, the 13-plex assay was also able to distinguish strains E/Bour and H/UW-43/Cx, due to distinct FLTs for targets IGS-102 in TMR and IGS-106 in ROX.

Eighty-eight purified DNAs from selected endocervical swabs consisting of 76 representative samples previously identified as “true” positives (samples 1 to 76 in Table 3) and 12 representative samples identified as “true” negatives (samples 77 to 88 in Table 3) for *Ct* infection were used as templates for rapid microfluidic multiplexed amplification. As expected, none of the representative negative samples were detected as positive for *Ct*. All profiles generated background signal with internal control, confirming that negative results were true negatives and not due to a failed PCR reaction. All 76 true positive clinical samples were confirmed to be *Ct*-positive using this assay.

Of these 76 samples, 75 were identified as urogenital and/or urogenital proctocolitis strains, and one (clinical sample 6, Table 3) was identified as noninvasive trachoma biovar (clade 3 or 4) as insufficient amplicons were generated for specific clade differentiation. The information from target IGS-105 indicates that this is not an LGV strain, and that from IGS-106 indicates that this is not a urogenital clade 2 strain; the clinical classification based on hAFLP type is reported in Table 3 as either clade 3 or 4. No ocular or LGV strains were identified in these endocervical clinical samples, all obtained from adolescent females at high risk for *Ct* infection. Twenty-one of these samples generated partial profiles, with at least 1 locus failing to amplify, but clade identification was still possible.

All positive samples were classified according to clade: 44 were urogenital samples (clade 2) with no Da strains identified, 7 were proctocolitis samples (clade 4), 24 had characteristics of both clades 2 and 4 (see below), and one was clade 3 or 4 (sample 6 as above). Interestingly, eight clinical samples (samples 51, 52, 53, 54, 55, 67, 68, and 69) generated a 345bp amplicon at the IGS-102 target (assigned as FLT iii in Table 3). This amplicon size was not expected based on *in silico* sequence alignments of available genome sequences that predicted only amplicon sizes of either 315 bp (FLT ii for clade 4) or 329 bp (FLT i for clade 2). Sanger sequencing of these amplicons revealed that the 345bp fragment observed at the IGS-102 locus was a result of a tandem duplication of 16 of the 329 bp FLT i sequence. S1 Fig (Supporting information) shows the sequence alignments in this target region and the location of the experimentally discovered 16bp tandem repeat.

The additional 7 blinded clinical samples were identified as positive for *Ct* (samples 89–95, Table 3*)*. Samples 89, 94, and 95 generated all the 12 of 13 expected peaks and FLTs for ocular trachoma, clade 3 with an hAFLP type 7 similar to known ocular *Ct* strains in Table 1. Sample 92 was also identified belonging to clade 3 despite the missing amplicon for IGS-101. For Sample 92, a new unique hAFLP type 25* has been assigned. Samples 90, 91, and 93 were classified as LGV, clade 1 and identified as serovar L_2_ based on FLTs from 12 or 13 loci. Two peaks (one at 418bp for LGV and one at 264bp for urogenital) were observed at IGS-107 locus in Sample 90; wherein in Sample 93, a urogenital peak (264bp) was seen instead of the expected 418bp LGV peak resulting in a new unique hAFLP type 26. The identification and typing with the 13-plex *Ct* assay was concordant with the data from *omp*A genotyping and MLST.

The experimentally determined FLTs from *Ct* strains (Table 2) and clinical DNA samples (Table 3), resulted in 33 hAFLPs. Five of the previously sequenced *Ct* reference strains and 18 of the blinded clinical samples had their hAFLP type assignments marked with asterisks, typically due to the absence of one amplicon. For example, strains B/TW5/OT and B/HAR-36 were assigned hAFLP type 10* since they were consistent with a 10 designation but did not amplify the IGS-101 locus. Ocular strain B-Har-36 (ATCC VR-573), urogenital clinical samples 48, 68, 69, 70, 75, and 76, ocular clinical sample 92, and LGV/L_2_ clinical sample 93 had unique hAFLP types. Four clinical samples (49, 50, 51, and, 52) were identified as urogenital or urogenital/proctocolitis but could not be assigned hAFLPs due to the absence of 3–7 amplicons.

### Limit of detection (LOD) assessment

Six representative strains from previously sequenced *Ct* reference strains were found in earlier tests to produce complete profiles with the assay (L1/440, L2/434, L3/404, H/UW-43/Cx, E/Bour, and C/TW-3/OT) at 100 genomic equivalents. To investigate *Ct* 13-plex LOD, we repeated the assay at 10 and 1 genomic equivalents. L3/404 generated a complete profile (12 FLTs) at 10 genomic equivalents; however, detection was still possible for the rest of strains with amplicons ranging from 4–8 loci. Both the multi-copy plasmid targets (*p*CT7 and *p*CT8) were detected in all strains. S2 Fig (Supporting information) shows a representative electropherogram of the proctocolitis *Ct* strain H/UW-43/Cx at 10 genomic equivalents. All 4 multi-copy detection targets in FAM were amplified, 3 out of 4 targets were detected in TMR and 1 out of 4 in ROX. At 1 genomic equivalent, 4 of 6 strains generated amplicons for 2–4 of the multi-copy targets.

### Assay specificity

**Fig 2:**
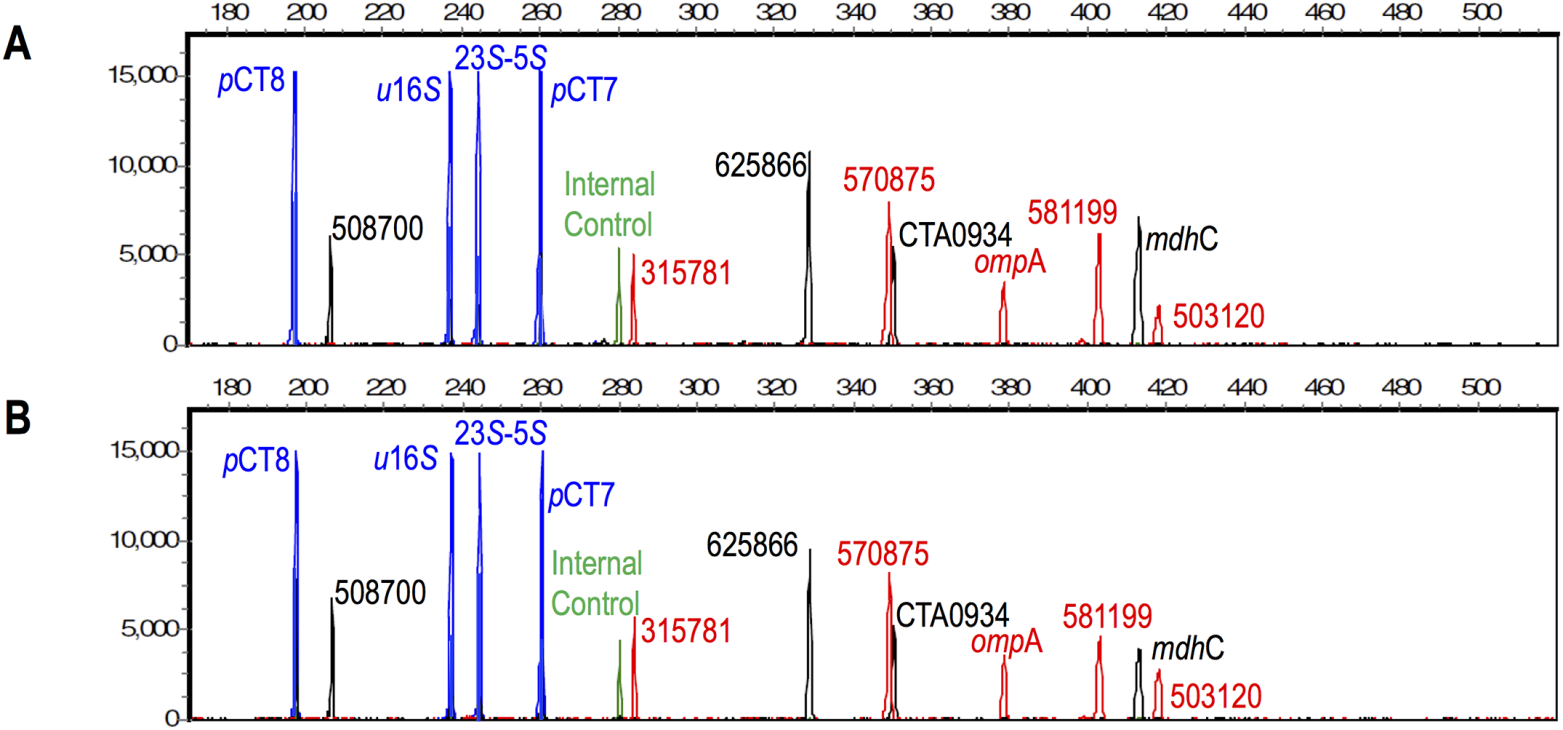
Specificity of the 13-plex *Ct* amplification assay using L1/440 Ct strain in the presence (A) or absence of *Neisseria gonorrhoeae* and human genomic DNAs as background DNAs (B). Strain L1/440 was tested at 100 genomic equivalents, and *Neisseria gonorrhoeae* and human genomic DNAs at 100,000 and 10,000 genomic equivalents, respectively. Background DNA had no significant effect on the characteristic *Ct* signature based on amplicon and internal control peak heights, and no nonspecific peaks were observed. X-axis: fragment size in bases; Y-axis: RFU.

Genomic DNA from 10 commensal/pathogenic microbial species (*Neisseria gonorrhoeae*, *Neisseria meningitidis*, *Neisseria flava*, *Neisseria perflava*, *Neisseria lactamica*, *Trichomonas vaginalis*, *Gardnerella vaginalis*, *Enterococcus faecium*, *Enterococcus faecalis and Candida albicans*) and from humans were added to amplification reactions containing *Ct* DNA. PCR reactions were carried out with 100 genomic equivalents of *Ct* in the presence of 100,000 genome equivalents of each microbial DNA and 10,000 genomic equivalents of human DNA. Fig 2 shows representative electropherograms from simultaneous amplification of 13 loci of *Ct* strain L1/440 in the presence of *Neisseria gonorrhoeae* and human genomic DNA (Fig 2A) and *Ct* DNA in the absence of background DNAs as positive control (Fig 2B). The 13-plex signature of the *Ct* genomes was unaffected in the presence of the background DNAs. None of the background species generated significant peaks.

## Discussion

The *Ct* multiplex PCR described here allows the simultaneous interrogation of 13 loci with numerous targets designed to detect and differentiate clinically significant strains. The assay enables differentiation of LGV strains from non-LGV strains and reduces the possibility that mutations, deletions, and recombination will result in false negatives secondary to primer target site elimination. Indeed, the value of this multiplex approach is well-illustrated by the failure in *Ct* detection previously observed with the Swedish variant in which a deletion at a primer-binding site negated identification by the single-locus Roche Amplicor NAAT [8, 25]. Growing evidence for *Ct* recombination and emergence of strains with indels at unpredictable locations support the implementation of multiplexed detection with redundant targets [14, 16].

The 13-plex *Ct* amplification assay confirmed the sequence-based biovar, clade and strain of all LGV and non-LGV samples tested excluding I/UW-12/UR which, when sequenced, generated results different from the known sequence of that strain, indicative of a sample labeling error. The LOD of the assay was shown to be 1–10 genomic equivalents. Chlamydia organism load varies by specimen type and site of sampling and majority of NAAT based studies found no association between load and signs and symptoms of the chlamydia infection [38]. In various studies, the number of chlamydia per genital swab ranged from fewer than 10 to greater than 10^6^, again with no association between chlamydial load and clinical symptoms [39, 40]. Chlamydial load was found to be higher in women (median load 10^5.6^/swab) than men (median load 10^3.5^/swab) [39]. Detection at levels of 1–10 *Ct* genomes suggests that the high multiplexicity of the assay allows sensitive, reliable, and robust *Ct* identification. The specificity of the assay was verified in the presence of 1,000-fold molar excess of pathogens and neighbor commensal species.

The high specificity of the 13-plex assay is important for reliable performance when using clinical samples typically containing DNA mixtures from human, commensal, and potentially pathogenic microorganisms in addition to *Ct*. No discordant results were detected between amplicon sizes generated from our assay and published whole genome sequence (WGS) data from previously sequenced strains, confirming assay reliability.

A high rate of recombination has previously been demonstrated across the *Ct* genome including the plasmid, most commonly between strains with tropism to the same tissue and occasionally between different biovars [26, 41, 42]. The inter-biovar recombination was illustrated by the isolation of an unusual LGV strain, L_2_c, where genomic sequencing revealed that it was a product of recombination between L_2_ and urogenital D strains [14]. Unexpectedly, the 13-plex assay identified 24 clinical specimens as having FLTs belonging to urogenital strains of both clades 2 and 4 and, within this group, nine samples that also have two distinct FLTs for the same target possibly indicating mixed infection by *Ct* belonging to different clades. In addition, the IGS-102 primer pair amplified a new FLT of 345 bp in eight clinical specimens. Genomic sequencing of these samples suggests that they could be a product of inter-clade recombination by horizontal gene transfer.

Most of the reference and clinical samples tested in our assay had an hAFLP signature that could be associated to a biovar and clade type: hAFLP type 4 was assigned to five *Ct* strains identified as LGV/ strain L_2_; hAFLP type 5 was assigned to 15 samples identified as proctocolitis/clade 4; hAFLP 7, 10, 10*, 11*, 25* were assigned to 10 total *Ct* samples identified as ocular trachoma/clade 3; and finally, a total of 55 samples identified as urogenital/clade 2 had 40 samples assigned as hAFLP type 6/6*, 7 samples assigned as hAFLP type 9/9*, and 4 samples assigned as hAFLP type 12. Taken together, our 13-plex assay can provide initial evidence of strain recombination in addition to detecting and genotyping *Ct* strains from clinical specimens. It would be valuable to conduct a wider study that would include clinical specimens from high risk patients acquired from various continents and from vaginal, rectal, and conjunctival swabs to evaluate the assay performance in genotyping global *Ct* strains from different anatomic sites. An additional application of our highly sensitive multiplexed test would be as a follow up test-of-cure to timely identify and appropriately treat persistent infections involving limited numbers of chlamydial organisms. Current available test-of-cure assays are not recommended for all individuals except pregnant women, partially due to insufficient sensitivity of such tests that can lead to false negative results [43].

Finally, we have recently developed a fully automated system that purifies DNA from a wide range of samples (including vaginal swabs), performs multiplexed PCR, and separates the resulting amplicons by gel electrophoresis [30, 31, 44]. Termed the ANDE (Accelerated Nuclear DNA Equipment) system and supported by the Department of Defense [45], it was designed for forensic short tandem repeat analysis outside the laboratory. The ANDE purification module requires approximately 10 minutes and the electrophoretic separation and detection module approximately 28 minutes. The fluorescent dyes utilized in the *Ct* assay were designed to use in the ANDE electrophoresis module, and the *Ct* amplifications described here were performed using the ANDE thermal cycler. The integration of the 22-minute microfluidic PCR assay described here into the ANDE system would allow a sample-in to result-out process of approximately one hour.

The availability of a rapid, sensitive, and specific POC diagnostic test for *Ct* may encourage broad screening of both symptomatic and asymptomatic individuals. Broad-based screening has the potential to facilitate early diagnosis and timely initiation of appropriate antibiotic treatment regimens, preventing severe complications and restricting further infection spread in the population. While useful to all individuals, such a fast and low cost POC test is of extreme importance, especially to the populations at high risk of infection.

## Supporting information

S1 Fig Sequence alignment at IGS-102 target region of Sanger sequencing results of amplification products from select clinical samples showing the experimentally discovered tandem repeat. Green arrow indicates the variable length region (deletion) that differentiates clades 2 and 4. Black arrows indicate the 1st and 2nd repeats. The 16bp duplication results in an amplicon size of 345bps. Clinical sample 5 represents a clade 4 sequence (315bp amplicon), clinical samples 17, 60, 63, 64, 66, 67 and 70 represent clade 2 sequences (329bp amplicon), and clinical samples 51, 52, 53, 54, 55, 56, 68, and 69 serve as examples of clade 2 sequences with tandem repeats (345bp). Select sequences of some known strains are aligned alongside the sequences of clinical samples to demonstrate that the new variant (with tandem duplication) originates from the variant present in clade 2 (without deletion and represented by sequences from serovars Ja, E, F, D, and Da aligned under the clinical samples) and not from clade 4 (with deletion and represented by sequences from serovars K, H, G, J, D, and Ia aligned above the clinical samples).
(TIF)

S2 Fig Representative electropherogram of the 13-plex *Ct* amplification assay using the proctocolitis *Ct* strain H/UW-43/Cx at 10 genomic equivalents showing amplicons from 8 of 13 loci. The internal control amplicon is JOE-labeled (green). X-axis: fragment size in bases; Y-axis: relative fluorescence units (RFU).
(TIF)

S3 Fig Sequence alignment at IGS-104 target region demonstrating the amplicon size difference among various representative variants. Green arrow indicates the variable region that defines FLT. Highlighted in yellow is the atypical strain L_2_b/48nl that has an LGV sequence signature different from the rest of the L_2_b strains. Black arrows indicate the primer binding sites.
(TIF)

S1 Table Sequences of *Ct* genomes used in alignments for primer design. (PDF)

S2 Table Sequences of primers used in the *Ct* 13-plex assay. (PDF)
