# Supplementary figures and images for: Rapid detection and strain typing of Chlamydia trachomatis using a highly multiplexed microfluidic PCR assay
Source: PLoS One. 2017 May 31;12(5):e0178653. doi: 10.1371/journal.pone.0178653 (PMC5451082; doi:10.1371/journal.pone.0178653)

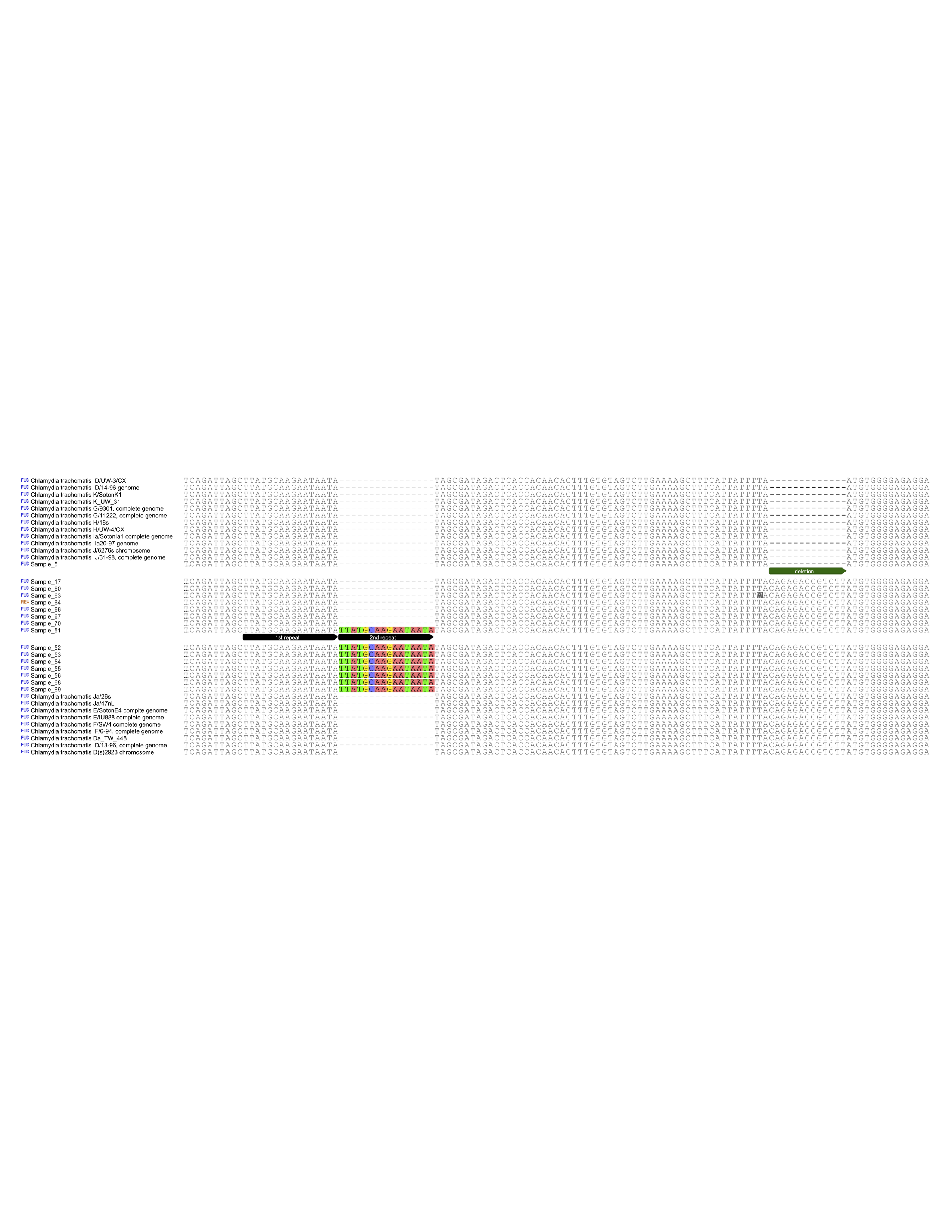

Supplement: S1 Fig — Green arrow indicates the variable length region (deletion) that differentiates clades 2 and 4. Black arrows indicate the 1st and 2nd repeats. The 16bp duplication results in an amplicon size of 345bps. Clinical sample 5 represents a clade 4 sequence (315bp amplicon), clinical samples 17, 60, 63, 64, 66, 67 and 70 represent clade 2 sequences (329bp amplicon), and clinical samples 51, 52, 53, 54, 55, 56, 68, and 69 serve as examples of clade 2 sequences with tandem repeats (345bp). Select sequences of some known strains are aligned alongside the sequences of clinical samples to demonstrate that the new variant (with tandem duplication) originates from the variant present in clade 2 (without deletion and represented by sequences from serovars Ja, E, F, D, and Da aligned under the clinical samples) and not from clade 4 (with deletion and represented by sequences from serovars K, H, G, J, D, and Ia aligned above the clinical samples). (TIF) [file pone.0178653.s001.tif]

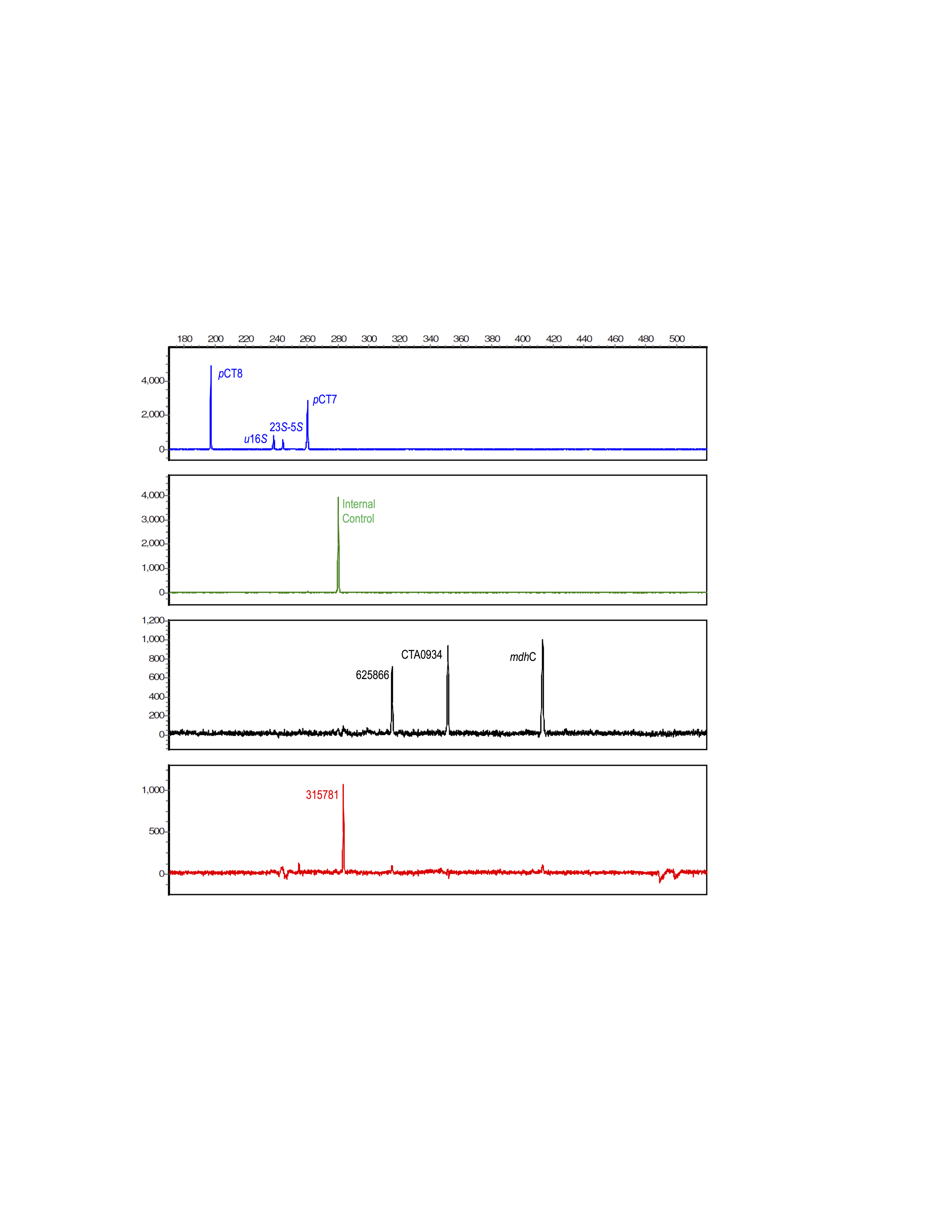

Supplement: S2 Fig — The internal control amplicon is JOE-labeled (green). X-axis: fragment size in bases; Y-axis: relative fluorescence units (RFU). (TIF) [file pone.0178653.s002.tif]

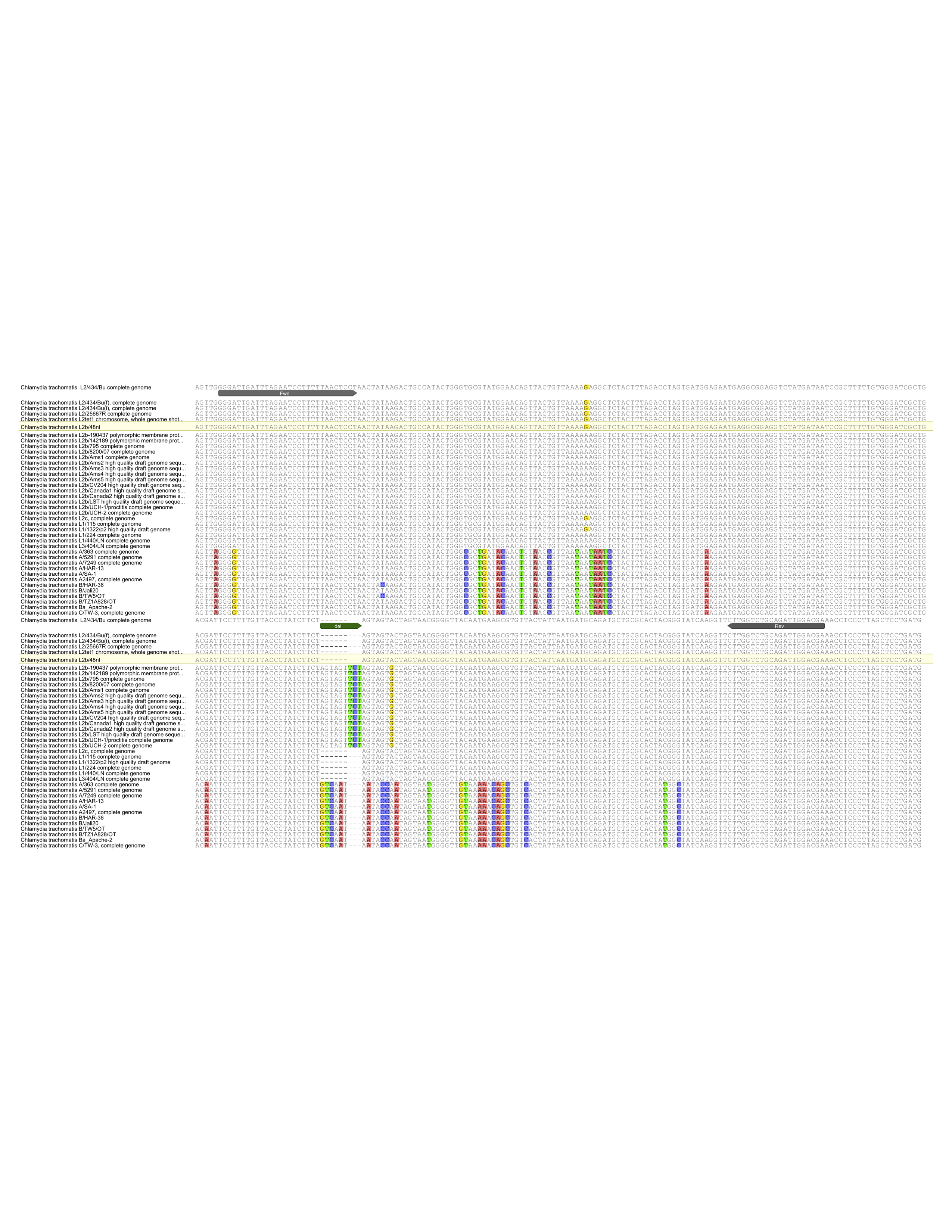

Supplement: S3 Fig — Green arrow indicates the variable region that defines FLT. Highlighted in yellow is the atypical strain L2b/48nl that has an LGV sequence signature different from the rest of the L2b strains. Black arrows indicate the primer binding sites. (TIF) [file pone.0178653.s003.tif]
