# Supplementary material for: Rapid detection and strain typing of Chlamydia trachomatis using a highly multiplexed microfluidic PCR assay
Source: PLoS One. 2017 May 31;12(5):e0178653. doi: 10.1371/journal.pone.0178653 (PMC5451082; doi:10.1371/journal.pone.0178653)
Supplement: S1 Table — (PDF) [file pone.0178653.s004.pdf]

S1 Table. Sequences of *Ct* genomes used in alignments for primer design.

Chlamydia trachomatis A2497 serovar A, complete genome
Chlamydia trachomatis A2497, complete genome
Chlamydia trachomatis A/363 complete genome
Chlamydia trachomatis A/5291 complete genome
Chlamydia trachomatis A/7249 complete genome
Chlamydia trachomatis A/HAR-13
Chlamydia trachomatis A/HAR-13, complete genome
Chlamydia trachomatis A/SA-1
Chlamydia trachomatis, strain: A/G-17/OT, ompA gene for major outer membrane protein, partial cds
Chlamydia trachomatis strain A/Har-1 major outer membrane protein (ompA) gene, complete cds
Chlamydia trachomatis strain B/Har-36 major outer membrane protein (ompA) gene, complete cds
Chlamydia trachomatis B/HAR-36
Chlamydia trachomatis B/Jali20
Chlamydia trachomatis B/Jali20/OT, complete genome
Chlamydia trachomatis B/TW5/OT
Chlamydia trachomatis strain B/TW-5 major outer membrane protein (ompA) gene, complete cds
Chlamydia trachomatis,strain: B/TW-5/OT, ompA gene for major outer membrane protein, partial cds
Chlamydia trachomatis B/TZ1A828/OT
Chlamydia trachomatis B/TZ1A828/OT, complete genome
Chlamydia trachomatis strain B/Tunis-864 major outer membrane protein (ompA) gene, complete cds
C.trachomatis major outer membrane protein serovar B gene, complete cds
Chlamydia trachomatis Ba_Apache-2
Chlamydia trachomatis strain Ba/Apache-2 major outer membrane protein (ompA) gene, complete cds
Chlamydia trachomatis, strain: Ba/AP-2/OT, ompA gene for major outer membrane protein, partial cds
Chlamydia trachomatis strain Ba/IU-FW0305 major outer membrane protein (omp1) gene, partial cds
Chlamydia trachomatis, strain: C/TW-3/OT, ompA gene for major outer membrane protein, partial cds
Chlamydia trachomatis strain C/TW3/OT major outer membrane protein 1 (omp1) gene, complete cds
Chlamydia trachomatis C/TW-3, complete genome
Chlamydia trachomatis strain C/TW-3 major outer membrane protein (ompA) gene, complete cds
Chlamydia trachomatis C-Ref
Chlamydia trachomatis strain C-Har32 major outer membrane protein (ompA) gene, complete cds
Chlamydia trachomatis strain CS-106/08 major outer membrane protein (ompA) gene, partial cds, Serovar C
Chlamydia trachomatis strain CS-110/03 major outer membrane protein (ompA) gene, partial cds, Serovar C
Chlamydia trachomatis strain CS-1228/06 major outer membrane protein (ompA) gene, partial cds, Serovar C
Chlamydia trachomatis strain CS-169/05 major outer membrane protein (ompA) gene, partial cds, Serovar C
Chlamydia trachomatis strain CS-19/08 major outer membrane protein (ompA) gene, partial cds, Serovar C
Chlamydia trachomatis strain CS-428/03 major outer membrane protein (ompA) gene, partial cds, Serovar C
Chlamydia trachomatis strain CS-434/03 major outer membrane protein (ompA) gene, partial cds, Serovar C
Chlamydia trachomatis strain CS-805/04 major outer membrane protein (ompA) gene, partial cds, Serovar C
Chlamydia trachomatis strain CS-820/07 major outer membrane protein (ompA) gene, partial cds, Serovar C
Chlamydia trachomatis strain CS-908/07 major outer membrane protein (ompA) gene, partial cds, Serovar C
Chlamydia trachomatis strain CS-936/06 major outer membrane protein (ompA) gene, partial cds, Serovar C
Chlamydia trachomatis strain CS-95/07 major outer membrane protein (ompA) gene, partial cds, Serovar C
Chlamydia trachomatis D1/91i
Chlamydia trachomatis D2/97i
Chlamydia trachomatis D/2s
Chlamydia trachomatis strain D2s major outer membrane protein gene, partial cds
Chlamydia trachomatis D/43nl
Chlamydia trachomatis strain D43nl major outer membrane protein gene, partial cds
Chlamydia trachomatis D/84s
Chlamydia trachomatis strain D84s major outer membrane protein gene, partial cds
Chlamydia trachomatis D/199nl
Chlamydia trachomatis D/210n
Chlamydia trachomatis D/2923
Chlamydia trachomatis D(s)2923 chromosome
Chlamydia trachomatis D(s)2923, whole genome shotgun sequence
Chlamydia trachomatis D/SotonD1 high quality draft genome sequence
Chlamydia trachomatis D/SotonD5 complete genome
Chlamydia trachomatis D/SotonD6 high quality draft genome sequence
Chlamydia trachomatis D/UW-3/CX
Chlamydia trachomatis D/UW-3/CX, complete genome
Chlamydia trachomatis strain D/UW-3 major outer membrane protein (ompA) gene, complete cds
Chlamydia trachomatis D-EC
Chlamydia trachomatis D-LC
Chlamydia trachomatis strain D/13-96, complete genome
Chlamydia trachomatis strain D/14-96 genome
C.trachomatis (D/B-120) omp1 gene for major outer membrane protein
C.trachomatis (D/B-185) omp1 gene for major outer membrane protein
C.trachomatis (D/IC-Cal-8) omp1 gene for major outer membrane protein
Chlamydia trachomatis strain D/ARG CA740 CT OmpA gene, partial cds
Chlamydia trachomatis strain D/LSU-EP212 major outer membrane protein 1 (OMP1) gene, complete cds
Chlamydia trachomatis strain D/LSU-PM12 major outer membrane protein 1 (OMP1) gene, complete cds
Chlamydia trachomatis strain D83s major outer membrane protein gene, partial cds
Chlamydia trachomatis, strain: Da/TW-448/OT, ompA gene for major outer membrane protein, partial cds
Chlamydia trachomatis Da_TW_448
C.trachomatis (Da/TW-448) omp1 gene for major outer membrane protein
C.trachomatis (strain Da/Ev-293) Omp1 gene
Chlamydia trachomatis E/5s
Chlamydia trachomatis E/93i
Chlamydia trachomatis E/150
Chlamydia trachomatis E/11023
Chlamydia trachomatis E/Bour
Chlamydia trachomatis E/C599 high quality draft genome sequence
Chlamydia trachomatis E/CS88(f) WGS:ANON01:Contig001, whole genome shotgun sequence
Chlamydia trachomatis E/CS88(i) WGS:ANOM01:Contig001, whole genome shotgun sequence
Chlamydia trachomatis E/SotonE4 complte genome
Chlamydia trachomatis E/SotonE8 high quality draft genome
Chlamydia trachomatis E/SW3 complete genome
Chlamydia trachomatis E/Sweden2
Chlamydia trachomatis strain E/12-94 genome
Chlamydia trachomatis, strain: E/UW-5/CX, ompA gene for major outer membrane protein, partial cds
Chlamydia trachomatis E/IU824 complete genome
Chlamydia trachomatis E/IU888 complete genome
Chlamydia trachomatis F/1
Chlamydia trachomatis F/11-96, complete genome
Chlamydia trachomatis F/38nL
Chlamydia trachomatis F/70
Chlamydia trachomatis F/70s chromosome
Chlamydia trachomatis F-Ref
Chlamydia trachomatis F/SotonF3 high quality draft genome sequence
Chlamydia trachomatis F/SW4 complete genome
Chlamydia trachomatis F/SW5 complete genome
Chlamydia trachomatis F/SWFPminus high quality draft genome sequence
Chlamydia trachomatis F/1-93, complete genome
Chlamydia trachomatis F/2-93 genome
Chlamydia trachomatis F/6-94, complete genome
Chlamydia trachomatis G1/1222
Chlamydia trachomatis G/9301
Chlamydia trachomatis G/9768
Chlamydia trachomatis G/11074
Chlamydia trachomatis G/SotonG1 complete genome
Chlamydia trachomatis G-Ref
Chlamydia trachomatis H/18s
Chlamydia trachomatis H/UW-4/CX
Chlamydia trachomatis strain H/UW-4 major outer membrane protein precursor (ompA) gene, complete cds
Chlamydia trachomatis H-clinical
Chlamydia trachomatis H-Ref
Chlamydia trachomatis strain ATCC VR-879=H/UW-43/cx major outer membrane protein (omp1) gene, partial cds
Chlamydia trachomatis strain I/UW12/Ur major outer membrane protein (omp1) gene, complete cds
Chlamydia trachomatis Ia/95i
Chlamydia trachomatis Ia/SotonIa1 complete genome
Chlamydia trachomatis Ia/SotonIa3 complete genome
Chlamydia trachomatis Ia-Ref
Chlamydia trachomatis strain Ia20-97 genome
Chlamydia trachomatis, strain: Ia/UW-202/Br, ompA gene for major outer membrane protein, partial cds
Chlamydia trachomatis isolate Ia/UW-202 major outer membrane protein 1 (omp1) gene, complete cds
Chlamydia trachomatis J_UW_36
Chlamydia trachomatis , strain: J/UW-36/CX, ompA gene for major outer membrane protein, partial cds
Chlamydia trachomatis J/6276 chromosome
Chlamydia trachomatis J/6276s chromosome
Chlamydia trachomatis J/6276tet1, complete genome
Chlamydia trachomatis strain J/27-97 genome
Chlamydia trachomatis strain J27s major outer membrane protein gene, partial cds
Chlamydia trachomatis strain J/31-98, complete genome
Chlamydia trachomatis Ja/26s
Chlamydia trachomatis strain Ja26s major outer membrane protein gene, partial cds
Chlamydia trachomatis strain Ja41nl major outer membrane protein gene, partial cds
Chlamydia trachomatis strain Ja47nl major outer membrane protein gene, partial cds
Chlamydia trachomatis Ja/47nL
Chlamydia trachomatis Ja-Ref
Chlamydia trachomatis isolate Ja/IU-37538 major outer membrane protein 1 (omp1) gene, complete cds
Chlamydia trachomatis K_UW_31
Chlamydia trachomatis strain K/UW-31 major outer membrane protein (ompA) gene, complete cds
Chlamydia trachomatis, strain: K/UW-31/CX, ompA gene for major outer membrane protein, partial cds
Chlamydia trachomatis K/SotonK1
Chlamydia trachomatis L1/115 complete genome
Chlamydia trachomatis L1/224 complete genome
Chlamydia trachomatis L1/440/LN complete genome
Chlamydia trachomatis L1/1322/p2 high quality draft genome
Chlamydia trachomatis strain L1/ARG CA043 CT OmpA (ompA) gene, partial cds
Chlamydia trachomatis strain L1/ARG CA696 CT OmpA (ompA) gene, partial cds
Chlamydia trachomatis strain L1/ARG CA699 CT OmpA (ompA) gene, partial cds
Chlamydia trachomatis strain L2/434/Bu complete genome
Chlamydia trachomatis L2/434/Bu(f), complete genome
Chlamydia trachomatis L2/434/Bu(i), complete genome
Chlamydia trachomatis L2/434/Bu
Chlamydia trachomatis L2/25667R complete genome
Chlamydia trachomatis L2tet1 chromosome, whole genome shotgun sequence
Chlamydia trachomatis L2tet1 NZ_ACUI01000001
Chlamydia trachomatis strain L2/T'ang=ATCC VR-577 major outer membrane protein (omp1) gene, partial cds
Chlamydia trachomatis, strain: L2a/UW-396/Nr, ompA gene for major outer membrane protein, partial cds
Chlamydia trachomatis strain L2a/UW-396 major outer membrane protein precursor (ompA) gene, complete cds
Chlamydia trachomatis L2b/48nl
Chlamydia trachomatis strain L2b48nl major outer membrane protein gene, partial cds
Chlamydia trachomatis L2b/795 complete genome
Chlamydia trachomatis L2b/8200/07 complete genome
Chlamydia trachomatis L2b/Ams1 complete genome
Chlamydia trachomatis L2b/Ams2 high quality draft genome sequence
Chlamydia trachomatis L2b/Ams3 high quality draft genome sequence
Chlamydia trachomatis L2b/Ams4 high quality draft genome sequence
Chlamydia trachomatis L2b/Ams5 high quality draft genome sequence
Chlamydia trachomatis L2b/Canada1 high quality draft genome sequence
Chlamydia trachomatis L2b/Canada2 high quality draft genome sequence
Chlamydia trachomatis L2b/CV204 high quality draft genome sequence
Chlamydia trachomatis L2b/LST high quality draft genome sequence
Chlamydia trachomatis L2b/UCH-1
Chlamydia trachomatis L2b/UCH-2 complete genome
Chlamydia trachomatis L2c, complete genome
Chlamydia trachomatis L2c major outer membrane protein gene, partial cds
Chlamydia trachomatis L3/404/LN complete genome
