# Supplementary material for: Rapid detection and strain typing of Chlamydia trachomatis using a highly multiplexed microfluidic PCR assay
Source: PLoS One. 2017 May 31;12(5):e0178653. doi: 10.1371/journal.pone.0178653 (PMC5451082; doi:10.1371/journal.pone.0178653)
Supplement: S2 Table — (PDF) [file pone.0178653.s005.pdf]

S2 Table. Sequences of primers used in the Ct 13-plex assay.

Target	Fluorescent Label	Forward primer (5'-3')	Reverse primer (5'-3')
*p*CT8	FAM	TGACCGATGTACTCTTGTAGAAAGTG	GTGGATTGGTTGATCGCCCAGAC
u16s	*FAM*	TAGTGTGTGAGGGGATAAATTGAGAG	GTTTAGCATCTATACTGGCCTGCATTCT
23s_5s	*FAM*	GCATTCTATTTCATTTGTGTTGTTAAGAGT	GTATTCGCGTCCTTCTTCGC
*p*CT7	FAM	GAATAGCTTTGTCTTTTTGGGATGATCG	GTTTCCTCTGAGAGTAATCTCGTTCATATTCG
IGS-101	TAMRA	CAACCAAGAAACTTTAACAACGTGTAAG	GTTCTGGATTTTCTTTATAGAGACACATTGAATCTAC
IGS-102	TAMRA	AGCGATAGACTCACCACAACACTT	GTTTCAGGTTCTTCCGGGAAGG
IGS-103	TAMRA	GGTGATATAATTGCACTCCCCGA	GTTTAGTTCTTAGTATAGGAAACCCTTCAAAAGA
*mdh*C	TAMRA	AGCTTTCTATTTTCTCTGGCTCATGG	GTAGCATCGCATGAAAGTTCTTTCTCAAT
IGS-104	ROX	GGGATTGATTTAGAATCCTTTTTAACTCCT	GTCGTCCAATCTGCAGACCAAG
IGS-105	ROX	CGTTCGCTACAACAAGAAACTCTC	CAAGAAGCCAAGGAAAGTGCG
*omp*A	ROX	GATTGAGCGTATTGGAAAGAAGC	GTTGCTGCAGCTCCATCCACTTG
IGS-106	ROX	ATCAGATTATGCAGATGTTTTAATTAGATCTCTT	GAGGTTCAAGGAAGTCCCTCCTAC
IGS-107	ROX	GTATAGACGCGAACAGGTTGTCT	GACTCCACTCACACCCAAACAAATC
Internal control	JOE	ACGTTTGGCATGGTTGTCGT	GTCGGATTGTACCCCTCGATGC
